# Influence of traditional markets on plant management in the Tehuacán Valley

**DOI:** 10.1186/1746-4269-9-38

**Published:** 2013-06-01

**Authors:** Yaayé Arellanes, Alejandro Casas, Anselmo Arellanes, Ernesto Vega, José Blancas, Mariana Vallejo, Ignacio Torres, Selene Rangel-Landa, Ana I Moreno, Leonor Solís, Edgar Pérez-Negrón

**Affiliations:** 1 Centro de Investigaciones en Ecosistemas, Universidad Nacional Autónoma de México, campus Morelia, Antigua Carretera a Pátzcuaro 8701, Col Ex Hacienda de San José de la Huerta, Morelia, Michoacán 58190, México; 2 Instituto Tecnológico de Oaxaca, Av. Ing. Victor Bravo Ahuja No. 125 esq. Calz. Tecnológico, C.P. 68030, Oaxaca de Juarez, Oaxaca, México; 3 Escuela Nacional de Estudios Superiores, Universidad Nacional Autónoma de México, campus Morelia, Antigua Carretera a Pátzcuaro 8701, Col Ex Hacienda de San José de la Huerta, Morelia, Michoacán 58190, México

**Keywords:** Barter, Domestication, Environmental management, Non-timber forest products, Plant management, Risk index, Traditional markets

## Abstract

**Background:**

The Tehuacán Valley, Mexico is a region with exceptionally high biocultural richness. Traditional knowledge in this region comprises information on nearly 1,600 plant species used by local peoples to satisfy their subsistence needs. Plant resources with higher cultural value are interchanged in traditional markets. We inventoried the edible plant species interchanged in regional markets documenting economic, cultural and ecological data and about their extraction and management in order to: (1) assess how commercialization and ecological aspects influence plant management, (2) identify which species are more vulnerable, and (3) analyze how local management contributes to decrease their risk. We hypothesized that scarcer plant species with higher economic value would be under higher pressure motivating more management actions than on more abundant plants with lower economic value. However, construction of management techniques is also influenced by the time-span the management responses have taken as well as biological and ecological aspects of the plant species that limit the implementation of management practices. Plant management mitigates risk, but its absence on plant species under high risk may favor local extinction.

**Methods:**

Six traditional markets were studied through 332 semi-structured interviews to local vendors about barter, commercialization, and management types of local edible plant species. We retrieved ethnobotanical information on plant management from ten communities in a workshop and sampled regional vegetation in a total of 98 sites to estimate distribution and abundance of plant species commercialized. Through Canonical Correspondence Analysis (CCA) we analyzed the amount of variation of management types that can be explained from socioeconomic and ecological information. A risk index was calculated relating distribution, abundance, economic value and management of plant resources to identify the most vulnerable species.

**Results:**

We recorded 122 edible plant species interchanged in the main regional markets. CCA explained significantly 24% of management variation, spatial distribution and plant parts used being particularly important in management decisions. The indeterminate 76% of variation suggests that management decisions depend on particular variables that are not explained by the ecological and socioeconomic factors studied and/or their high variation in the context at the regional scale. The risk index indicated that management was the factor that mostly influences decreasing of risk of interchanged plant species. We identified *Clinopodium mexicanum, Pachycereus weberi, Dasylirion serratifolium, Disocorea* sp., *Ceiba aesculifolia, Neobuxbamia tetetzo, Lippia graveolens, Litsea glaucescens, L. neesiana, Jatropha neopauciflora, Agave potatorum* and other agave species used for producing mescal among the more endangered plant species due to human pressure, their relative scarcity and limited or inexistent management.

**Conclusion:**

Spatial distribution and plant parts used are particularly meaningful factors determining risk and influencing management actions on edible plant species interchanged in the region. Limited or inexistent management may favor extinction of local populations under risk. Local management techniques synthesize knowledge and experiences crucial for designing sustainable management programs. Traditional management techniques supported by ecological information and environmental management approaches could make valuable contributions for sustainable use of plant species, particularly those becoming economically important more recently.

## Background

Interchange was in the past and currently is in Mesoamerica a key institution that allows peoples from a particular region to obtain products from diverse ecological and cultural areas to complement their livelihoods [1,2]. Chroniclers of the Spanish conquest of Mexico [3-5] described important Pre-Columbian markets called “***tianquiste***” in Náhuatl (the Aztec language) and “plazas” in Spanish which made possible reconstructing how these markets were, trade routes of particular plant products and interchange nets in the whole region and between Mesoamerica and other areas of the New World [6]. Some important plant resources documented in historical sources are species native to the Tehuacán-Cuicatlán Valley (ahead shortly called Tehuacán Valley) which were and are part of the diet of regional people [7-9]. Prominent are the cases of ‘***tempesquistle***’ (*Sideroxylon palmeri*), ‘***pochote***’ (*Ceiba aesculifolia*), prickly pears and pitayas or ‘***nochtli***’ (fruits of *Opuntia* spp. *Stenocereus* spp., *Polaskia* spp., *Escontria chiotilla*, among others), agave flowers and stems (*Agave* spp.), and dozens of other species [7,9,10]. A number of both native and non-native plant species are still interchanged in the regional ***tianquiste***[11-13]; some of them are resources important from pre-Columbian times and others are products that became commercially important more recently.

Interchange influences rhythms at which plant products are extracted from ecosystems and also may influence decisions about its management form, but this latter aspect has been scarcely studied [11,12,14]. This topic is particularly important to be studied in the Tehuacán Valley since this region has been widely recognized as a meaningful area for understanding processes of domestication of plants and origins of agriculture [7]. Studying current relations between interchange and management of local biodiversity in this region makes possible to analyze human pressures and motives for managing and domesticating plants, which in turn may be helpful to model possible scenarios of plant management in the past [12,15].

### The case study in the Tehuacán Valley

Archaeological research in the Tehuacán Valley documented that humans have been present in the region for more than 10,000 years, and found among the oldest signs of management and domestication of plants in the New World [7,16,17]. In addition, ethnobotanists have documented current forms of plant management involving domestication of native plant species associated to both silvicultural and agricultural systems [9,12,18,19]. Plants diversity is high in the Tehuacán Valley; Dávila et al. [20] reported nearly 3,000 vascular plant species, and such diversity coexists with an also high cultural richness represented by numerous rural communities of eight indigenous groups (Nahua, Popoloca, Cuicatec, Ixcatec, Chocho, Mazatec, Mixtec and Chinantec [9]). The close relationship between local peoples and natural resources of the region for thousands of years [7,21] have generated a deep traditional knowledge and diversified strategies of management of plants, animals and ecosystems. In this region, ethnobotanists have documented one of the richest inventories of useful plants of Mexico, with over 1,600 plant species used for different purposes by local peoples [9,22]. Nearly 36% of these plant species receive some kind of management [12], and most of the managed species (nearly 60%) are native to the area, coexisting with their wild relatives occurring in local forests. A gradient of management of edible plant species has be documented to exist in the region [9,22,23]. Of a total of 339 edible plant species registered in the whole area, 257 (nearly 75%) are under some management type other than simple gathering.

The spectrum of management practices, in addition to simple gathering includes: (1) systematic gathering, involving communitarian agreements for planning strategies, social organization, specialized techniques and tools, and different harvesting intensities, (2) in situ management techniques, including tolerance or let standing, enhancing or promotion and special care of particular species and/or phenotypes of some species preferred by people in areas disturbed for different purposes, mainly agroforestry systems and natural forest areas, (3) agricultural systems which include a gradient of management intensity, from slash and burn agriculture in small parcels, terraces, and intensive agro-industrial agriculture in irrigated areas [9,12,15,24,25]. Through management people modify and adapt the availability of plant resources according to their needs [8,26] with the purpose of guaranteeing amount and quality of resources. All this information suggests that the Tehuacán Valley is a main center of plant management in Mesoamerica, ideal for documenting how decisions and techniques for managing resources are constructed and how on-going processes of domestication of plants occur [19,27].

Traditional markets involve interchange that influences mechanisms of social articulation of peasant societies [28]. The term “traditional” used for characterizing these markets involves aspects such as: i) pre-Columbian origin and features similar to those of conquest chronicles’ descriptions, ii) specific ***tianquiste*** or plaza days, once or twice per week, iii) interchange of products either through cash or by barter, and iv) presence of sellers from diverse areas, mainly women carrying plants harvested at low scale in their home gardens, parcels or forests around their villages [11]. Barter is a particularly important characteristic of traditional markets [29,30] through which people interchange natural resources through maize seeds, products elaborated with maize, fruits, flowers, roots and other plant products [13,31-33]. In the Tehuacán Valley people interchange plant products used as food, medicine, firewood, raw materials for handcrafts, supplies for construction and ornamental plants [11]. This study focuses on edible plants. Previously, the cases of ‘***pochote***’ (*Ceiba aesculifolia* subsp. *parvifolia*) [18,31], ‘mescal agave’ (*Agave potatorum* Zucc.) [34], ‘***tempesquistle***’ (*S. palmeri*) [32], columnar cacti fruits: ‘***xoconostle***’ (*Stenocereus stellatus*) [8], ‘***tetechas***’ (*Pachycereus weberi*) [13,33], and ‘***jiotilla***’ (*E. chiotilla*) [13,33] have been documented and illustrated that sale of edible plants may represent significant monetary incomes.

Economic aspects of traditional markets have been well documented [29,30], but little is known about pressures of markets on plant populations, and how these factors may motivate plant management. To analyze these issues, we explored the following questions: 1) what richness of edible plant species is interchanged in traditional markets of the Tehuacán Valley?, 2) which are main socioeconomic and ecological factors influencing management of edible plants?, and 3) which edible plant species are more vulnerable to risk due to high trade and/or inadequate management and/or scarcity? Because of the high biocultural diversity of the Tehuacán Valley we expected to find a high plant species richness interchanged in local traditional markets. We presumed that salespeople have developed rhythms and strategies of extraction and other technological responses to meet the demand for certain plant products. It was therefore expected that edible plants interchanged in markets have higher risk and consequently receive more management than other useful plants registered in the region. Such patterns should be particularly clear in plant species whose distribution and abundance is restricted. We therefore hypothesized that scarcer plant species with higher economic value would be under higher pressure motivating more management actions than on more abundant plants with lower economic value. However, not all plant species endangered are necessarily managed because time for developing techniques has been short or because biological characteristics, such as difficult propagation or maintenance, may limit success of management. We then also expected that not managed native plants under increasing commercial demand are particularly endangered, especially those with restricted distribution and abundance. This latter could be the situation of species becoming commercial plant resources more recently and for which management techniques have not been still developed. Traditional forms of constructing decisions and management techniques are valuable bases for designing sustainable management strategies for regional biodiversity conservation.

## Methods

### Study area

**Figure 1:**
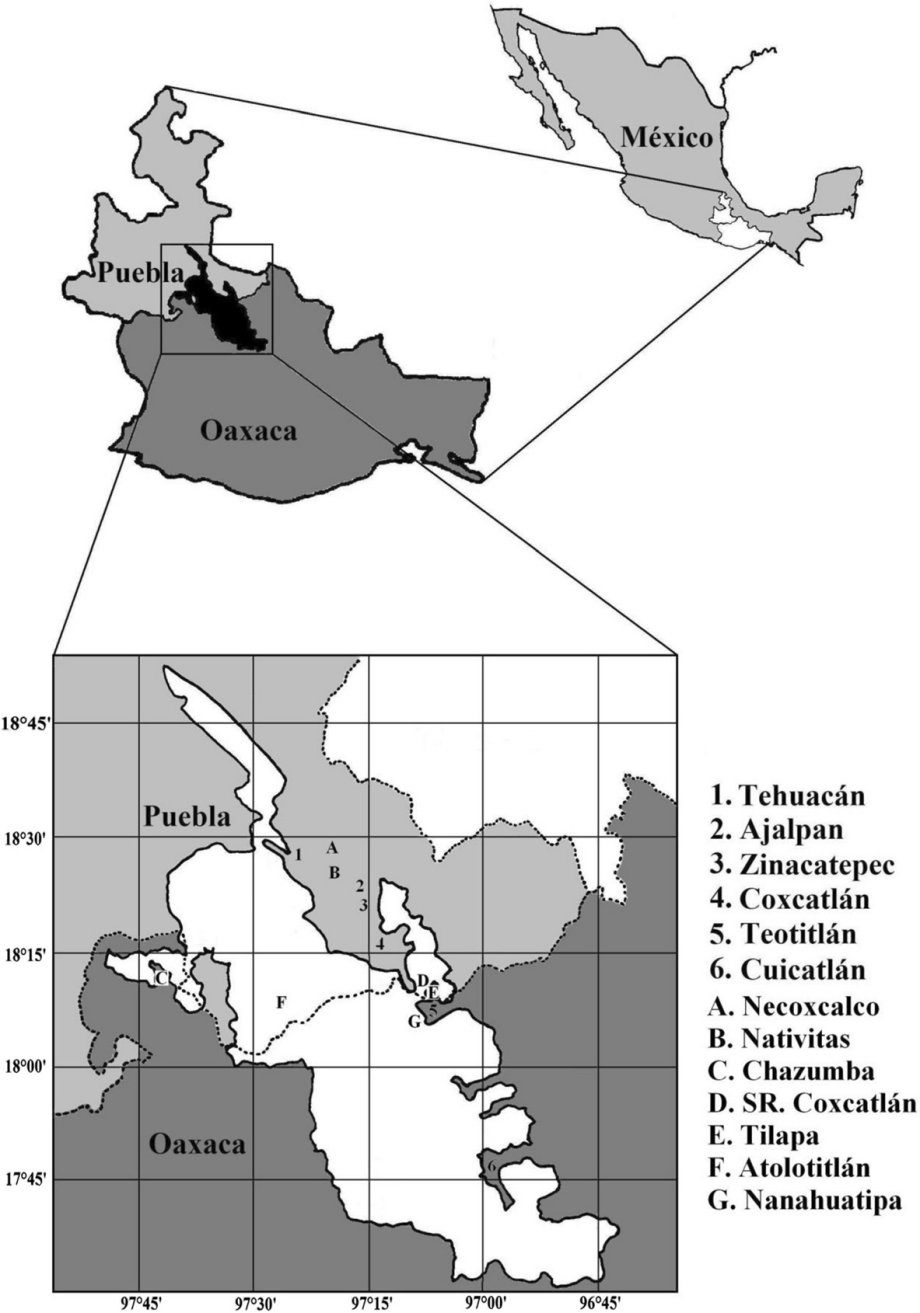
**Study area.** Numbers indicate the location of the traditional markets studied in the Tehuacán-Cuicatlán Valley. Letters indicate some places where sellers come from.

Information on edible plants was collected in the main traditional markets of the Tehuacán Valley: Ajalpan, Zinacatepec, Coxcatlán and Tehuacán in the state of Puebla, and Teotitlán and Cuicatlán in the state of Oaxaca (Figure 1). This region, extending ca. 10 000 km^2^ represents a complex physiographic mosaic with different climate types, predominantly semi-arid with an average temperature of 21°C and an average annual rainfall of 400 mm [35]. Ecological diversity is represented by a high beta diversity with 36 types of plant associations [36,37] grouped in the following main vegetation types: i) columnar cacti forests; ii) lowland woody forests (at elevations below 1800 m); iii) highland woody forests (at elevations higher than 1900 m); iv) riparian vegetation; v) thorn-scrub forest and, vi) schlerophyllus-scrub forest.

### Data collection in traditional markets

Information about use, management and origin of edible plants was documented through direct and semi-structured interviews with vendors in the six markets mentioned. Nearly 80 sellers arrived from different localities to a market and established stands on “plaza” days (Table 1). The number of stalls was determined from participatory observations and interviews with four local public employees who recorded the number of seller at each market.

**Table 1:** **Sociocultural and environmental information of market villages studied**[11]

**Market Village**	**Village total population**	**Ethnic groups represented in market customers and sellers**	**No. of different surrounding villages that use the market**	**Market day(s) and Type of market**^**1**^
San Juan Bautista Cuicatlán	3,920	Cuicatec, Ixcatec, Mestizo, Mazatec	7	Friday and Saturday (municipal ≈ 40 stands)
Teotitlán de Flores Magón	7,390	Mazatec, Nahua, Mestizo	27	Wednesday and Sunday (municipal ≈ 80 stands)
Coxcatlán	5,600	Nahua, Mixtec, Mestizo	7	Sunday (local ≈ 40 stands)
San Sebastián Zinacatepec	14,500	Nahua, Mixtec, Mestizo	18	Thursday (municipal ≈ 80 stands)
Ajalpan	24,800	Nahua, Mixtec, Popoloca, Mazatec, Mestizo	24	Wednesday and Sunday (microregional ≈ 180 stands)
Tehuacán	238, 200	Nahua, Mixtec, Mazatec, Cuicatec, Chocho, Popoloca, Mestizo	29	Saturday (regional ≈ 250 stands)

We visited the markets of Ajalpan, Cuicatlán, Zinacatepec and Tehuacán eight times (a total of 205 interviews), we visited the market of Teotitlán 21 times (107 interviews) and five times the market of Coxcatlán (a total of 20 interviews). For conducting interviews and botanical collections we asked permits from the Biosphere Reserve authorities and from the Authorities of the Environment Ministry of Mexico. For studying the mentioned markets we asked permits to the particular authorities of the respective municipalities, and the personal authorization of people interviewed. We interviewed sellers speaking Náhuatl, Mazatec, Mixtec, Cuicatec, and Spanish, with the help of local people for translation. Semi-structured interviews were carried out with sellers about the role of edible plant species in their subsistence. Main topics of the interviews were: i) the names of plant resources in Spanish and indigenous languages, ii) provenance of sellers and the edible plants commercialized, iii) ecological status of plants (wild, weedy, ruderal or domesticated), v) management intensity (if plant species were simply gathered, had incipient in situ management such as tolerance, enhancing and special care, or if plants were cultivated in agricultural systems), vi) sales volume, vii) other uses for the plant besides its edibility, viii) plant parts utilized (fruit, root, leaf, flower or the entire plant), ix) spatial availability (regional distribution and abundance) and season when the resource is available, and x) economic value, as to whether it is interchanged for other products (barter) or if the transaction is with money (commercialization). Botanical specimens obtained in the markets were determinate at the herbarium of the Instituto de Ecología A. C., Mexico (XAL).

### Regional information database

A database of the edible plant species of the region with ecological, socio-economic and management information (see aspects in Additional file 1) was constructed to: 1) analyze how management types are related to ecological and socioeconomic variables, and 2) construct a risk index evaluating how much plant species permanence is compromised because of high demand in markets, its relation to their availability and the adequate or inadequate management. Information supporting these analyses was obtained from three different matrices with ecological, socioeconomic, and management data respectively for 122 edible plant species registered in the traditional markets studied (Additional file 1). The matrix with ecological data was constructed with average, percentages or frequencies and summed information (for instance for summarizing information about density, frequency, parts used, uses, among others, see Additional file 1 for ecological, socio-economic and management data) of 98 sites of vegetation samplings conducted throughout the region by researchers authoring this study (Table 2) as well as ecological information obtained from market interviews. The matrix with socioeconomic data on edible plant resources was constructed with information from a total of 332 interviews to sellers in the traditional markets studied; average data were calculated per plant species. The management data matrix was based on information on plant management systematized by Blancas et al. [12], through workshops carried out by ethnobotanists working in the region, as well as additional information recorded through interviews in markets. Information about management was obtained in 10 case studies in the region. Ethnobotanists co-authoring this study participated in workshops sharing their information and analyzing information recorded in markets. Studies at communitarian level were conducted with the permits of authorities of the biosphere reserve, the municipalities, and the communities studied. We considered as “native” those plant species with natural populations occurring wild and or weedy in the region, and as “introduced” those plant species from other regions of Mexico and other parts of the world.

**Table 2:** Sociocultural and environmental information of the villages where the 98 vegetation samplings were taken

**Village**	**Ethnic group**	**Total population**	**No. of plots**	Vegetation types*	**Elevation range (m)**	**Reference**
Chimalhuacan, Coyomeapan	Nahua	189	2	Thorn-scrub forest (2)	1740	[51]
Aticpac, Coyomeapan	Nahua	153	3	Woody forest at elevations below 1,800 m (3)	1080	[51]
Ahuatlán, Coyomeapan	Nahua	413	4	Highland woody forest (1), agroforestal system in highland woody forest (3)	1980	[52]
Santa María Coyomeapan	Nahua, Mazatec	937	3	Highland woody forest (1), agroforestal system in highland woody forest (2)	1950-2000	[52]
Yohuajca, Coyomeapan	Nahua	304	1	Highland woody forest (1)	2422	[52]
San Luis Atolotitlán	Mestizo	922	19	Agricultural system(2), Agroforestal system in Columnar cacti forest (3),Thorn-scrub forest (3), Highland woody forest (3), Woody forest at elevations below 1800 m (6), riparian vegetation (2)	1880-2300	[53]
San Rafael Coxcatlán	Nahua, Mestizo	261	2	Woody forest at elevations below 1800 m (2)	900-1000	[54]
Santa María Ixcatlán	Ixcatec, Mestizo	573	17	Highland woody forest (8), Woody forest at elevations below 1800 m (2), riparian vegetation (1), schlerophyllus scrub forest(2), agricultural system (2), grassland (2)	1750-2200	[55]
San Lorenzo Pápalo	Cuicatec	583	29	Highland woody forest (13), Woody forest at elevation below 1800 m (2), agricultural system(8), riparian vegetation (2), Agroforestal system in highland woody forest(4)	1400-2460	[52,56]
Santiago Quiotepec	Mestizo	176	14	Columnar cacti forest (8), riparian vegetation (2), agricultural system (4)	550-700	[57]
San José Tilapa	Nahua, Mestizo	1,977	2	Woody forest elevation below 1800 m (2)	900-1000	[54]
Teotitlán de Flores Magón	Nahua, Mazatec, Mestizo	7,598	2	Woody forest elevation below 1800 m (2)	900-1000	[54]

*As characterized by Valiente-Banuet et al. [36] Numbers in parentheses indicate plot samplings of each type of vegetation.

### Data analysis

#### a) Variation partitioning of management

**Figure 2:**
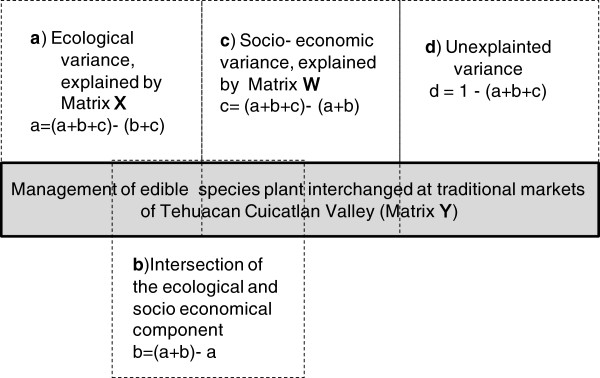
**Partition of the variation of a response matrix Y between Ecological (matrix X) and Socioeconomic (matrix W) explanatory variables.** Fraction (b) is the intersection of the ecological and socioeconomic components of the management variation. The length of the horizontal line corresponds to 100% of the variation of Y. Modified from Boccard et al. 1992.

Using R software [38,39] and based on Boccard et al. [40]’s model we conducted a Canonical Correspondence Analysis (CCA) to evaluate the amount of variation of management data that can be explained from ecological and socioeconomic information. The model was constructed considering only plant species with complete information (a total of 105 species). The socioeconomic and ecological variations are not unrelated since the spatial-temporal availability of a particular species in the market is related to environmental factors, and therefore confusion about causes and effects would be expected. For this reason we used the proposal by Boccard et al. [40] through three matrices partitioning the variation: Matrix Y contains the response variables (management data matrix), matrix X is the set of explanatory ecological variables; and matrix W is the set of explanatory socioeconomic variables (Table 3, Figure 2). Through this method we conducted several CCA combining the sets of explanatory variables: 1) CCA only for matrix Y, 2) CCA for matrix Y vs. matrix W, 3) CCA for matrix Y vs. matrix X, 4) CCA for matrix Y vs. matrices W + X. The total constrained Eigen value of each analysis was tallied to identify how much of the management matrix is explained by ecologic and socioeconomic data. This method allowed the division of CCA variation into four parts: *a)* Ecological data, which is the fraction of management variation that can be explained by ecologic data independently of socioeconomic data, *b)* Socioeconomic + ecological data, *c)* Socioeconomic data which is the fraction of management variation that can be explained by socioeconomic data independently of ecologic data, and, *d)* Undetermined data o fraction of management variation explained neither by ecological nor by socioeconomic data (Figure 2). For each of these analyses, the sum of all canonical eigenvalues divided by the sum of all (unconstrained) eigenvalues, gives the corresponding fraction of the variation explained by the analysis. To evaluate the significance of the models for each CCA we performed a permutation test to evaluate significance of: a) the whole model, b) management explained by ecological variables and 3) management explained by socioeconomic variables.

**Table 3:** Matrices used in the partial canonical ordination (CCA)

**Matrices**	**Variables**	**Description**
**Management (Response Matrix Y)**	Ecological Status*	Conditions of habitat of a plant species, whether wild, weed or ruderal or domesticated
	Management types*	Conditions of a plant’s management, whether gathered, tolerated, promoted, protected or cultivated
	Management Systems*	Spatial systems where a plant is found, ranging from natural vegetation to intensive cultivation
	Number of Uses	Sum of uses for each species, plus edible use, such as medicinal, forage, ornamental, etc.
**Ecological (Matrix X)**	Spatial distribution	Spatial availability or number of plots where a species was present on 98 plots (Table 2)
		Presence in one o more of six vegetation types grouped by Valiente-Banuet et al. 2000 [36].
	Temporal distribution	Temporal availability of the months than edible plants presents at markets.
	Used Part Index	Plant used index (based on Pieroni 2001 [58].
	Life cycle	Annual or perennial
**Socioeconomic (Matrix W)**	Number of markets	Market presence, between 6 markets and 1 market
	Number of stalls	Average market stall were species plant is present
	Average price	Average price of a plant species in all markets
	Sales Volume	Total sales volume for a day in all markets
	Interchanged ways	Transaction realized by barter and/or money or just by maize.

* These variables were recategorized and were considered as another variable because some plant species had more than one value in this category. Values were summed in the recategorized variables.

#### b) Risk index

In order to assess edible plant risk, we considered ecological, socioeconomic and management factors for 57 native edible plant species with complete ecological and socio-economic data (Table 4). Each variable was divided by the maximum value of its category, the highest risk value being close to 1 and the lowest one close to 0. We designed the risk index with higher values indicating higher risk. In those cases in which values of variables go in opposite directions (asterisk of Table 4), such as management factors, the value was reset to 1, in order to modify the range.

**Table 4:** Factors included in the risk index of native edible plant species interchanged

**Indicator**	**Variables**	**Least risk**	**Most risk**	**Values range (minimum to maximum)**
**Ecological factors**	Spatial distribution*	Wide distribution	Restricted distribution	98 plot sites to 1 plot
	Temporal distribution*	Many months	One month	12 months to 1 month
	Used Part Used Index (as Pieroni 2001)	Flowers and bud harvest	Whole aerial plant used	1.0 (as bark) to 3.0 (as whole aerial parts)
	Life cycle	Perennial	Annual	Least intensive used (perennial 1), most harvesting (annual 2)
**Socioeconomic factors**	Number of markets	1 market	6 markets	1 to 6 markets
	Number of stalls	One stall	Many stalls	1 to 9
	Average price*	Lowest price (calculated per 1 kg)	Highest price (calculated per 1 kg)	$1.90 to $308.5
	Sales Volume	Low	High	0.19 kg to 661.90 kg
	Interchanged ways*	Many methods of interchange	Just with money	2 (interchanged by barter and money), 1 (interchanged just with money).
**Management factors**	Ecological Status*	Domesticated	Wild	Domesticated (3), Weed or ruderal (2), wild (1)
	Management types*	Cultivated	Gathered (Foraged)	Cultivated (5) Protected (4), Promoted (3), Tolerated (2), Gathered (1)
	Management Systems*	Intensive System	Natural Vegetation	Intensive system (5), Homegardens (4), Agroforestal System (3), Secundary Vegetation (2), wild vegetation (1)
	Number of Uses*	More uses	Less Uses	

For management factors were recategorized and were considered as another variable because some plant species had more than one value in this category. Values were summed in the recategorized variables.

The risk index (*R*_*i*_) for each species was calculated as the product of ecologic (*fa*_*i*_), socioeconomic (*fb*_*i*_) and management (*fc*_*i*_) factors as follows:

$$R_{i}=\left(\sum_{a=1}^{A}\frac{fa_{i}}{\max\left(\mathrm{fa}\right)}+\sum_{b=1}^{B}\frac{fb_{i}}{\max\left(\mathrm{fb}\right)}+\sum_{c=1}^{C}\frac{fc_{i}}{\max\left(\mathrm{fc}\right)}\right)$$

Where: *i* = species *i*, *a* = ecological features (factors or data), *b* = socioeconomic data, *c* = management data, *A* = Total ecological data, *B* = Total socioeconomic data, *C* = Total management data. *R*_*i*_ values go from 0 to unity, as each addend in the sum is a proportion. The format of *R*_*i*_ allows its graphical representation in a triangle graph, where the relative importance of each addend is easily seen.

## Results

### Edible plant species richness, interchanged in markets

A total of 122 edible plant species of 45 botanical families were recorded in the stands of traditional markets. The richest families were: Solanaceae (13 species), Fabaceae (13), Cactaceae (11), Rosaceae (8) and Cucurbitaceae (7), which include nearly half of all edible plant species interchanged in the regional markets. The richest plant families except Rosaceae and Lamiaceae have a higher proportion of native species (Additional file 1). Agavaceae and Lauraceae are represented only by native species and Brassicaceae includes only introduced species. Proportion of introduced edible plant species is slightly higher than native species (63 and 59 species, respectively). The number of edible species increased proportionally with the size of the market (Table 1).

**Figure 3:**
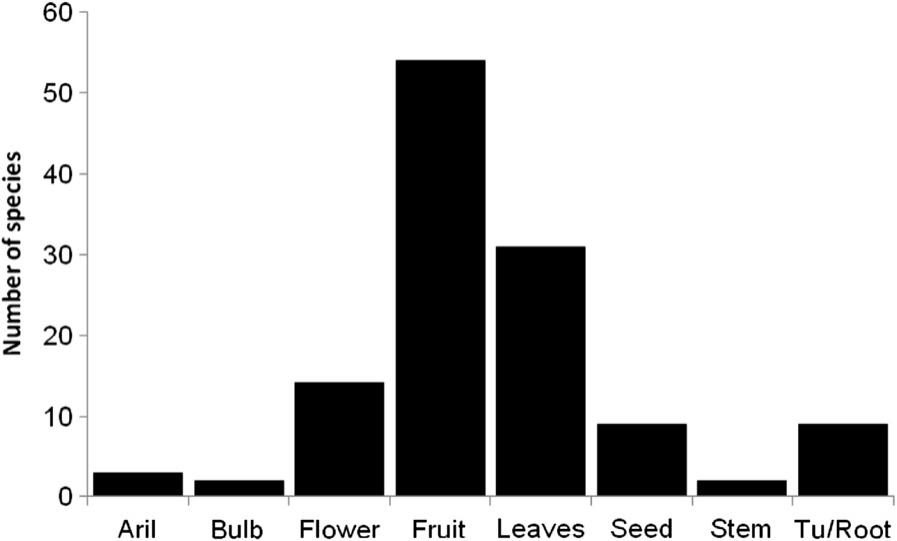
Edible species numbers in relation to the part consumed in the Tehuacan-Cuicatlán Valley markets.

Barter is a common system of interchange used in traditional markets. It is a common transaction in five of the six studied markets, where we recorded that 22% to 78% of the products that are offered can be interchanged through this method. Ajalpan was the market with the greatest practice of barter, and Cuicatlán was the only market with no barter recorded. The most interchanged parts of edible plants are fruits, leaves and flowers (Figure 3). Most of the structures interchanged were found in particular seasons. For instance, in April and May it is possible to find fruits of the columnar cactus *Myrtillocactus geometrizans* and between May and August those of *Stenocereus pruinosus.* In general, leaves consumed as greens were more common in the markets than fruits or flowers. Most plant species producing edible leaves are cultivated, which allows people to offer these products in markets during continuous periods of time, and are plant parts with relatively low prices; these are for instance the cases of *Amaranthus hybridus, Porophyllum macrocephalum, P. linaria* and *Sonchus oleraceus*. Leaves that are used as spices have higher prices than greens and are required in smaller portions; these are for instance the cases of *Lippia graveolens* and *Thymus vulgaris*. The most expensive plant parts are seeds from native species such as *Pachycereus weberi, Jatropha neopauciflora, Pinus cembroides* and *Ceiba aesculifolia* subsp. *parvifolia*. Seeds of the native plant species *Apodanthera aspera* were the most expensive of all edible products of native plants recorded in the markets.

Edible plant species recorded generally have more than one use and management type. We recorded a total of 48 edible plant species obtained through simple gathering; 32 species receive forms of incipient management, and 88 are under agricultural management. Most plant species under simple collection and incipient management are native; these are for instance the cases of *Agave potatorum, Yucca periculosa, Escontria chiotilla, Neobuxbaumia tetetzo, Stenocereus stellatus, S. pruinosus, Ceiba aesculifolia*. Fourteen edible plant species under agricultural management are native, including: *Chenopodium berlandieri, C. ambrosioides*, *Stenocereus stellatus,* and *S. pruinosus*. Use and management types of edible plant species may differ between localities and was different in each market studied. Nearly 90% of vendors interviewed are gatherers of forest products or cultivators of edible plants. Market activity with edible plants was a highly gendered activity: nearly 95% of sellers are women.

### Management types influenced by ecological and socioeconomic factors

Partitioning analysis explains 24% of the variation of management. This variation can be explained mainly by ecological factors (16%), while socioeconomic data explain 5.8% and both, ecological and socioeconomic factors explain 0.9%. Most variation of the model is undetermined (76%), and the whole model is statistically significant (Table 5a). Permutation test for CCA under reduced model of management factors and ecologic and socioeconomic factors shows that the first three CCA are significant (Table 5b). The whole model permutation test shows two significant variables: spatial availability and plant part used (Table 5c).

**Table 5:** Overall fit, importances of axis and explanatory variables of CCA model of how ecologic and economic factors influence management of edible plants in Tehuacán valley

**a) Overall fit**				
	Inertia	Proportion	F	Pr(>F)
Total	0.15524	1		
Constrained	0.03659	0.23568	2.8985	0.001
Unconstrained	0.11865	0.76432		
**b) Importance of axis**				
	Chisq	F	Pr(>F)	
CCA1	0.0277	23.3615	0.001	
CCA2	0.0044	3.7319	0.03	
CCA3	0.0037	3.1228	0.0466	
CCA4	0.0007	0.6191	0.62	
**c) Importance of variables**				
	Chisq	F	Pr(>F)	
%Plot	0.0186	14.748	0.001	
%Vegetation Types	0.002	1.5651	0.257	
%Month	0.0016	1.2291	0.319	
Part Used Index	0.0033	2.5864	0.081	
Life Cycle	0.0021	1.7011	0.199	
%Markets	0.0011	0.9067	0.465	
Average Market Stall	0.0029	2.2877	0.111	
Average Price	0.0019	1.508	0.25	
Volumen sold total	0.0013	1.0569	0.382	
Interchanged	0.0018	1.3962	0.306	

a) General fit of the model, b) Relative importance of CCA axis, c) Significance of explanatory variables on management.

### Risk index

**Figure 4:**
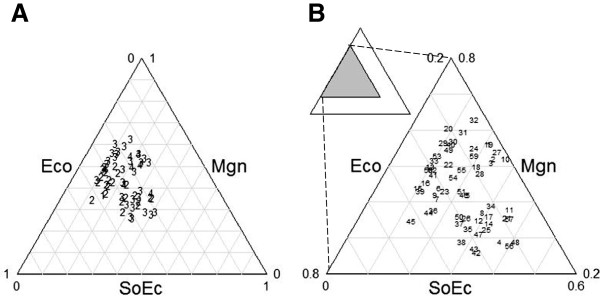
**Relative contribution of ecological (Eco), socioeconomic (soEco) and management (Mgn) factors on the risk index value (*R*).** In both panels the arrows show the direction of lecture of each factor. For example, in panel B species plant circled 45 (*Renealmia alpina*) has a risk value 2 (intermediate risk) in panel A. This species has the same coordinates in both panels (Eco = 0.55, SoEc = 0.13, Mgn = 0.35). **A**. Absolute magnitudes of index risk values. Larger numerical values denote higher risk index values. Each plant species is represented with a number code: 1) low risk (0.32 < *R* ≤ 0.39); 2) intermediate risk (0.40 < *R* ≤ 0.49); 3) intermediate-high risk (0.5 < *R* ≤ 0.59); 4) high risk (0.6 < *R* ≤ 0.67). **B.** Species identity in the triangle. Acronyms = 1: *Agave potatorum,* 2: *A.*sp.1, 3: *A.*sp.2*,* 4*: Amaranthus hybridus,* 5: *Apodanthera aspera,* 6*: Byrsonima crassifolia,* 7: *Capsicum annuum,* 8: *C. annuum var. annuum,* 9: *C. annuum* var. *aviculare,* 10: *Ceiba aesculifolia* subsp. *parvifolia,* 11: *Chamaedorea tepejilote,* 12: *Chenopodium ambrosioides,* 13: *C. berlandieri,* 14: *Crataegus pubescens*, 15: *Crotalaria pumila,* 16: *Cucurbita mostacha,* 17: *C. pepo,* 18: *Cyrtocarpa procera,* 19: *Dasylirion serratifolium,* 20: *Dioscorea sp.,* 21: *Escontria chiotilla,* 22: *Ferocactus latispinus,* 23: *Inga jinicuil,* 24*: Jatropha neopauciflora,* 25: *Leucaena esculenta,* 26: *L. leucocephala,* 27: *Lippia graveolens,* 28: *Litsea glaucescens,* 29: *L. neesiana,* 30: *Myrtillocactus geometrizans,* 31: *Neobuxbaumia tetetzo,* 32: *Pachycereus weberi,* 33: *Peperomia peltilimba,* 34: *Persea americana,* 35: *Phaseolus coccineus,* 36: *P.* sp., 37: *P. vulgaris,* 38: *Physalis philadelphica,* 39: *Phytolacca icosandra,* 40: *Pinus cembroides,* 41: *Pithecellobium dulce,* 42: *Porophyllum linaria,* 43: *P. macrocephalum,* 44: *Prunus serotina,* 45: *Renealmia alpinia,* 46: *Clinopodium mexicanum,* 47: *Sechium edule,* 48: *Sideroxylon palmeri,* 49: *Solanaceae* sp*.,* 50: *Solanum lycopersicum,* 51: *S. nigrescens,* 52: *S. sp.,* 53: *Spathiphyllum cochlearispathum,* 54: *Spondias mombin,* 55: *S. purpurea,* 56: *Stenocereus pruinosus,* 57: *S. stellatus,* 58: *Witheringia solanacea,* 59: *Yucca periculosa.*

For each species, risk index value obtained was plotted in a triangle (Figure 4). Each axis of the triangle is a factor (management, socioeconomic and ecological factors) with values between 0 and 1 and each plant species is represented by a triangle in Figure 4a. Index values had a range between 0.32 to 0.67. None of the species recorded had a risk index value close to 1. Index values were divided in four range intervals which correspond to numbers of Figure 4a, such as 1 = 0.32-0.39 or low risk, 2 = 0.40-0.49 intermediate risk, 3 = 0.5-0.59 intermediate-high risk, and, 4 = 0.6-0.67 high risk. Each factor contributes differently for each plant species. In general, most species are located in the left middle of the triangle which indicates that socioeconomic factors contribute little to the risk index whereas management is the factor with the highest contribution. Management influences the relationship with risk, so that when there is management, the risk decreases. Zoom from Figure 4b shows distribution of plant species. *Prunus serotina* (1), a native widely cultivated species has the lowest risk value whereas the highest was recorded in *Clinopodium mexicanum* (4), a wild plant species with high demand in the market, under over-extraction and with no management involved. Analysis of distribution of plant species shows that contribution of each factor to the risk index is not proportional and each species requires a particular analysis. For instance, *Pachycereus weberi, Diospyros* sp*.* and *Neobuxbamia tetetzo* have intermediate-high (3) risk index values and management factor have an important contribution, but socioeconomic factors have low contribution (Figure 4a). In general, the maximum contribution of socioeconomic factors to a species risk was not higher than 40%. These effects can be appreciated in the cases of *Amaranthus hybridus, Stenocereus pruinosus* and *Sideroxylon palmeri* with intermediate risk values. Ecological factors were important in *Renealmia alpinia* and *Prunus serotina*, but these species had low risk index values.

## Discussion

Our study found a high floristic richness of species of edible plants that are interchanged by small-scale vendors in traditional markets. In the individual stalls of six markets we registered the presence of 34% of all edible plant species documented for the whole Tehuacán Valley [9,22]. Almost half of the edible plant species recorded are native, and most of them are managed in one or more forms. The analysis of the relation of socioeconomic and ecological factors allows an evaluation of risk endangering future maintenance of plant species, their influence motivating plant management, and the role of management decreasing risk.

A similar percentage of edible plants species sold in the traditional markets is managed compared to all plants managed in the region (74.5% in the markets and 75.81% in the whole region [12,22]). Management actions may differ among species or among the markets and areas studied, a pattern similar to that reported by Blancas et al. [12]. For instances, in the market of Teotitlán vendors indicated that they did not manage the supplies of the native species *Agave potatorum*, while one of the development projects for the western region, in San Luis Atolotitlán, involves the propagation of this species [41]. In the case of *Ceiba aesculifolia* subsp. *parvifolia* management has been documented suggesting incipient domestication in Coxcatlán, Tilapa and San Rafael [18,31], but market vendors from Tilapa and San Rafael affirmed that they do not perform any activity that promotes the presence of this species. Therefore, each species has different types of management across local or regional boundaries. This information indicates that it is necessary to conduct particular analysis and documenting more detailed information to determine the differences and similarities of local and regional management. However, the experience of management existing for a particular species in a particular area of the region could be extraordinarily important when designing conservation policies at regional scale.

**Figure 5:**
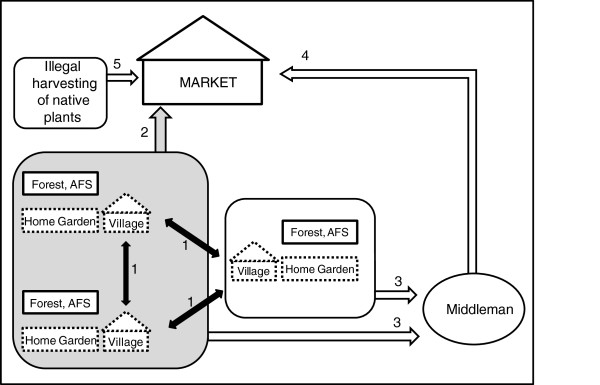
**Trade routes of edible plants species at Tehuacán-Cuicatlán Valley.** Edible plant species came from the forest, agroforestry system (AFS) and from home gardens and are produced at low scale. Gray area corresponds to the analysis of this study. 1 = interchange between village inhabitants through barter or commercialization, 2 = plants taken to the market by village inhabitants and exchanged for money or other products, 3 = Plants exchanged for money or other products by middlemen inside villages, 4 = Plants collected at villages and offered directly by middlemen or wholesalers who exchange only for money at the market.

Using regional information on management and ecology of plants species the partitioned CCA analysis allowed understanding the factors that motivated people to manage edible plants. This model showed that 24% of management variation can be explained, with ecological factors being the most meaningful. Therefore, factors such as spatial distribution, presence of a specific species of plant, and the plant part used are important in understanding the type of management response and the time period in which people make decisions about the actions that take place to use an edible plant. Although our model explains only 24% of the management variation, it is statistically significant (Table 5a). The remaining 76%, that is the unexplained variance, can be understood by considering that management actions are different between markets and between localities. Therefore, management depends on local or specific variables that are not explained just by ecologic and socioeconomic factors, and because of the high variation of ecological and cultural contexts at regional level; there are particular management conditions and contexts characterizing the matrixes. Our study documented the socioeconomic information of six markets and we know the routes by which native plants arrive at those markets (Figure 5). Based on this information, we can say that the traditional “plazas” provide significant information about the actions that may influence the management of edible plants interchanged in the region.

In general, occurrence or not of management practices and the type of management practiced on a plant species was one of the most influential factors in estimating the risk in permanence of edible species interchanged. In fact, the three factors which we controlled for analyzing potential risk to species – that is, ecological, socioeconomic and management factors – were far from equally balanced (Figure 5). Interpretation of risk index requires a careful analysis; the meaning of the numerical index is helpfully complemented by the graphic representation. The numerical risk index helped us to understand the degree of risk, whereas the triangle of the graphic representation helped us understanding the relative weight of each factor’s contribution to the index. These triangles show which of the three is the most important factor in risk of a particular species. One important thing to notice is that species with higher risk index values are the 14 native plant species that are exclusively gathered; these are the cases of Solanaceae species, as well as *Peperomia peltilimba, Lippia graveolens, Dioscorea* sp.*, Pachycereus weberi, Ceiba aesculifolia, Litsea glaucescens, Litsea neesiana, Agave* sp. 1, *Dasylirion serratifolium, Agave* sp. 2, *Clinopodium mexicanum,* and *Jatropha neopauciflora*. This is not to say that those plants that are managed in some way are not at risk. Although there is a great overlap between plants interchanged in market and those which are managed, for certain native species, the high amount of products commercialized evidences the intensive extraction, and lack of management of these plants could affect the local populations. An example is the case of *Ceiba aesculifolia* (pochote), which is collected intensively in San Rafael Coxcatlán and San José Tilapa. In those localities, nearly 20,000 fruits per household are collected every reproductive season [18,31] and in one “plaza” day we documented the sale of almost 160 kg of cooked seeds. Interviews with local vendors revealed an increasing scarcity of other species such as (i) *Neobuxbamia tetetzo* in San Antonio Nanahuatipam and in Coxcatlán, (ii) *Jatropha neopauciflora* in Chazumba, (iii) *Dasylirion serratifolium* in Ajalpan, San Esteban Nocoxcalco and Santa María Teopoxco, (iv) *Myrtillocactus geometrizans* in Santa María Nativitas and (v) *Pachycereus weberi* in Coxcatlán. The vendors of each of these products said that every year they had to walk further into the woods to find these species, and it was progressively more difficult to find them. Such increasing scarcity may also influence costs that provide a negative cycle of feedback for the species. We found that almost 27% of native plants cost around $3 USD or more per kg, while only 8.4% of introduced edible plant costs up to $3 USD per kg. That is, products from native plant species are more expensive than those from introduced species.

Commercialization of plant products in the traditional “plazas”, is an indicator of a particular level of ecological risk, but these plazas are not the only ways that native plants arrive at the markets. The risk to native plants and their conservation could also be related to the routes where they are sold. Other ethnobotanical studies conducted in the region indicate that the markets we studied receive only part of the edible species plant regionally interchanged as shown in Table 6. Nevertheless, markets involve interchange of more edible plant species than other forms of commercialization of the region. Whitaker and Cutler [42], documented 83 edible plant species in the markets of Tehuacán, Ajalpan and Teotitlán, 28% of them being native to México (56% of the New world, 42% Old world and 2% pan-tropical, Table 6). Pardo [13] reported 73 species of edible plants, 75% of them being native to the region and although 51 species were exchanged in markets, only 31 species were shared with the present study. However, it is necessary to take into account that the studies mentioned were carried out several years ago and that the research effort was different; therefore, the data are not necessarily comparable.

**Table 6:** Ethnobotanical studies conducted in the Tehuacán-Cuicatlán Valley which provide information on edible plant species

**Localities**	**Ethnofloristic richness**	**Edible plants**	**Commercialized edible plants**	**Location where sold**	**References**
Tehuacán, Ajalpan, Teotitlán *markets*	83	83	N/E	Markets	[42]
San Rafael Coxcatlán *homegardens*	233	69	3	At village	[59]
Zapotitilán, Coxcatlán, San Antonio Cañada, Cuicatlán, *markets and localities*	72	72	38 with money 28 by barter 11 by “order”	Markets and at village	[13]
Santiago Quiotepec *Locality*	(266) 203 useful	74	17	At and between villages around and to resellers	[33]
San Pedro Nodón y Santiago Jocotipac, *localities*	(264) 110 useful	21	5	At and between villages around and to resellers	[60]
San Luis Atolotitlán *locality*	280 useful	44	6	At and between villages	[53]
San Lorenzo Pápalo *locality*	(520) 367 useful	84	6	At and between villages around and to resellers	[56]
Zapotitlán de las Salinas *locality*	(298) 288 useful	82	n/e	n/e	[61]
Santa María Tecomavaca most common edible plants *locality*	20	20	9 (potentially)	At and between villages	[23]
Coxcatlán *Homegardens*	314	N/E	10 (with money and barter)	At and between villages	[62]
Tehuacán *Market*	30	30	30 (with money and barter)	Markets	[63]
San Luis Atolotitlán *Agroforesty system*	122	23	11 (with money and barter)	At and between villages	[26]
Zapotitlán de las Salinas *Locality*	58	27	n/e	n/e	[64]
Ixcatlán *Locality*	(482) 376	68	4	At village	[55]

Most markets studied functioned at least partially on a system of barter. In five of the six markets studied, this type of transaction was observed, and depending on the market, between 22% and 78% of the products offered could be exchanged through barter. The use of barter is not frequent when the resources offered have a price of more than $5 US dollar per kg, that is, when they “are expensive,” or when fruits or vegetables are of prime quality. This type of interchange is more frequent with products that are frequently found in the market, with fruits and vegetables of lower quality, or with low cost products. Traditional markets of Ajalpan, San Sebastián Zinacatepec, and Coxcatlán share a regional particularity of the exchange of plant products through maize. Those localities have a high level of economic marginalization [43]. Corn is not offered by the low-volume vendors, but it is highly appreciated as a product of interchange because it is the main staple food in Mexico.

For people, the traditional markets are ways to obtain incomes through the commercialization and/or barter of products, and it is also a backdrop for the exchange of knowledge and management techniques. According to Arizpe [44] and Arellanes and Casas [11], the main importance of these markets are the social, economic, and cultural roles in the communities they take place, but our study also emphasizes their ethnobiological importance in knowledge of numerous local plant species that are used, and in the management actions taken for their conservation. Information of the conservation status of each species requires socio-ecological studies identifying localities where plant populations are endangered because of their scarcity, human pressure and inadequate forms of management. Sellers recognize that plants they remove directly from their ecosystems require adequate forms of management to avoid their extinction, but socio-economic pressures determine that for some cases large amounts of edible plant products are extracted and brought to the markets without any plan or action for their replacement. Among the factors that the sellers recognize as pressures favoring the extensive extraction of resources are: (i) the difficult economic situation (poverty) and need of obtaining monetary incomes; (ii) the increasing number of gatherers because of the absence of other economic alternatives for local people; (iii) the limited access to the resources (since the sites from which they are taken are part of the Tehuacán-Cuicatlán biosphere reserve), which influences looting, illegal sales, (iv) the lack or scarcity of fair and strict communitarian rules governing the use of common resources; (v) the restricted spatial and temporal availability of edible plant species and products; and (vi) climate change affecting the behavior, health and availability of species interchanged.

The risk to conservation of native plants is multifactorial. The high pressure exerted by humans is a principal cause of over-exploitation of certain non-timber forest products and the rates of increasing transformation of the earth ecosystems [45]. Their overuse can affect the species at different scales of their organization, from functions, life cycle, survival, and up to the availability of propagules [46]. Promoting sustainable growth would be an essential action to ensure the continuing presence of a species, but such sustainable use is not only related to the plants’ commercial potential. In fact, the sustainability of resources, whether commercialized or not, is the result of multiple factors at the communitarian and regional levels and differ throughout space and time. Shackleton [45] asserts that the existence of markets for forest resources does not guarantee the conservation of a species. González-Insuasti et al. [47] demonstrated that for one community in the Tehuacán-Cuicatlán Valley, an important factor in the survival of species is type of land use. Undoubtedly, social organization at all scales will be indispensable in constructing agreements about the utilization and preservation of the land and its resources. Documenting and implementing local people’s traditional knowledge and management techniques nay have a significant impact on governmental programs for sustainable use of native resources [48]. The diversity of ecosystems and human cultures in the Tehuacán Valley, and the long history of their interaction have contributed to the broad spectrum of forms of use and management of edible plants species. In this context, the traditional markets studied are a biocultural memory of the region [49,50].

The existence of various human cultures, their distinct culinary costumes, the variable range of socioeconomic situations, the differential availability and forms of access to resources, help to understand that management of plant resources at each location depends on multiple factors that need to be analyzed particularly for species and communitarian contexts. The better our understanding of the use of plants destined for the market, the more precise would be our capacity for identifying the most effective strategies for the conservation of a species and the ecological richness.

Our study identified those interchanged edible plant species under more risk or more vulnerable, such as *Ceiba aesculifolia* subsp. *parvifolia*, *Jatropha neopauciflora*, *Dasylirion serratifolium*, and columnar cacti *Neobuxbamia tetetzo*, *Pachycereus weberi* and *Myrtillocactus geometrizans*. These plant species require specific ecological studies to know their current situation, abundance, reproductive biology, demography, among other topics. Also, specific studies about their management and socioeconomic factors affecting their populations. For each plant species mentioned is required taking actions to encourage the conservation according to the magnitude of the problem, hopefully constructing programs generated jointly between villagers, government, social organizations and scholars for protection and sustainable use of plant resources.

## Competing interests

The authors articulate than they have no competing interest.

## Authors‘ contributions

YA main author, involved in the study design, conducting of interview, field work, literature review and general data collection and systematization, wrote the first draft and concluded the final version this paper. AC main coordinator-supervisor of the research project; contributed with original data and the designing of all the researches providing the information for the current analysis; participated in fieldwork, systematization and analysis of data and reviewed several drafts of the manuscript. AA designed part of study methods, participated in compilation and analysis of literature review, reviewed and modified the first and final draft versions of the manuscript. EV suggested performed statistical analysis, teaming with YA and AC. JB, MV, IT, SR, AIM, EPN, LS, CL conducted ethnobotanical and ecological studies of plant resources in different communities and regions of the Tehuacán Valley, participated in several workshops providing original information for the data analysis. All authors read and approved the final manuscript.

## Supplementary Material

Additional file 1 **Appendix 1.** Edible plant species documented in traditional markets in the Tehuacán-Cuicatlán Valley. Appendix2. Data used in Partial Canonical Ordination Analysis. Asterisks show raw data not used in the analysis. Appendix 3. Database used by Risk Index. Higher values indicated higher risk, the highest risk value is closer to 1 and the lowest one closer to 0. Appendix 4. Description of partial canonical analysis variables used in the database of 105 edible plant species. Appendix 5 Description of partial canonical analysis variables used in the database of 59 edible plant species. F factor 1: 1 = Manag*e*ment, 2 = Ecological, 3 = Socioeconomic. Click here for file
