# Supplementary material for: Influence of traditional markets on plant management in the Tehuacán Valley
Source: J Ethnobiol Ethnomed. 2013 Jun 1;9:38. doi: 10.1186/1746-4269-9-38 (PMC3698157; doi:10.1186/1746-4269-9-38)
Supplement: Additional file 1 — Appendix 1. Edible plant species documented in traditional markets in the Tehuacán-Cuicatlán Valley. Appendix2. Data used in Partial Canonical Ordination Analysis. Asterisks show raw data not used in the analysis. Appendix 3. Database used by Risk Index. Higher values indicated higher risk, the highest risk value is closer to 1 and the lowest one closer to 0. Appendix 4. Description of partial canonical analysis variables used in the database of 105 edible plant species. Appendix 5 Description of partial canonical analysis variables used in the database of 59 edible plant species. F factor 1: 1 = Management, 2 = Ecological, 3 = Socioeconomic. [file 1746-4269-9-38-S1.doc]

**Appendix 1** Edible plant species documented in traditional markets in the Tehuacán-Cuicatlán Valley. Collector’s names were converted to letters such as: AVB=Alfonso Valiente-Banuet, EPN=Edgar Pérez-Negrón, ER=Erandi Rivera, IT=Ignacio Torres, LS=Leonor Solís, RL=Rafael Lira, SR=Selene Rangel-Landa, YA=Yaayé Arellanes, YE=Yven Echeverría.

| **No.** | **Family** | **Species** | **Common name** | **Voucher specimen** |
| --- | --- | --- | --- | --- |
| 1 | Alliaceae | *Allium cepa* L. | Cebolla | ER177 |
| 2 | Alliaceae | *Allium sativum* L. | Ajo | Photo record |
| 3 | Amaranthaceae | *Amaranthus hybridus* L. | Quelite | YA693, YA739, EPN384 |
| 4 | Anacardiaceae | *Cyrtocarpa procera* H.B. & K. | Chupandio | EPN49, YE310 |
| 5 | Anacardiaceae | *Mangifera indica* L. | Mango | Photo record |
| 6 | Anacardiaceae | *Spondias mombin* L. | Obo | LS32 |
| 7 | Anacardiaceae | *Spondias purpurea* L. | Ciruela | Photo record |
| 8 | Annonaceae | *Annona cherimola* Mill. | Chirimoya | EPN144, LS342 |
| 9 | Annonaceae | *Annona reticulata* L. | Anona | EPN404, LS119 |
| 10 | Apiaceae | *Coriandrum sativum* L. | Cilantro de monte | YA593, YA741 |
| 11 | Apiaceae | *Petroselinum sativum* Hoffm. | Perejil | ER72 |
| 12 | Araceae | *Spathiphyllum*  *cochlearispathum* (Liebm.) Engl | Oloxoxhitl | YA700 |
| 13 | Arecaceae | *Chamaedorea tepejilote* Liebm.  ex Mart. | Tepejilote | YA637, 701 |
| 14 | Asparagaceae | *Agave potatorum* Zucc. | Cacayas, Toshmezcalt | YA726, SR403 |
| 15 | Asparagaceae | *Agave* sp. 1 | Cacayas | YA673, 750 |
| 16 | Asparagaceae | *Agave* sp. 2 | Cacayas | YA633, 759 |
| 17 | Asparagaceae | *Yucca periculosa* F. Baker | Izote | YA715 |
| 18 | Asteraceae | *Lactuca sativa* L. | Lechuga |  |
| 19 | Asteraceae | *Porophyllum linaria* (Kunth) DC. | Pápalo | YA572, YA621, SR357 |
| 20 | Asteraceae | *Porophyllum macrocephalum*  DC. | Pipicha | YA613, YA743 |
| 21 | Asteraceae | *Sonchus oleraceus* L. | Quelite | YA729 |
| 22 | Bignoniaceae | *Parmentiera edulis* DC. | Cuajilote | Photo record |
| 23 | Bixaceae | *Bixa orellana* L. | Achiote | Photo record |
| 24 | Brassicaceae | *Brassica campestris* L. | Nabo | YA626, YA747 |
| 25 | Brassicaceae | *Brassica oleraceae* subsp.  *spacephala* L. | Col | YA703 |
| 26 | Brassicaceae | *Raphanus sativus* L. | Rábano | SR44 |
| 27 | Brassicaceae | *Rorippa nasturtiumaquaticum*  (L.) Hayek | Berro | YA653, YA686, YA749 |
| 28 | Cactaceae | *Acanthocereus subinermis*  Britton & Rose | Nopal de cruz | YA636 |
| 29 | Cactaceae | *Escontria chiotilla*  (F.A.C. Weber) Rose. | Jiotilla | IT451 |
| 30 | Cactaceae | *Ferocactus latispinus*  Britton & Rose | Biznaga | IT454 |
| 31 | Cactaceae | *Hylocereus undatus*  (Haw.) Britton & Rose | Pitahaya | IT452 |
| 32 | Cactaceae | *Myrtillocactus geometrizans*  Britton & Rose | Garambuyo | YA709 |
| 33 | Cactaceae | *Neobuxbaumia tetetzo*  (F.A.C. Weber) Backeb. | Tetecha | YA714, IT457 |
| 34 | Cactaceae | *Opuntia auberi* Pfeiff. | Cocochitos | YA632, YA753 |
| 35 | Cactaceae | *Opuntia* sp. | Tuna | Photo record |
| 36 | Cactaceae | *Pachycereus weberi*  (J.M. Coulter) Backeb. | Semilla de cardón | Photo record |
| 37 | Cactaceae | *Stenocereus pruinosus*  (Otto) F. Buxb. | Pitayas | IT462 |
| 38 | Cactaceae | *Stenocereus stellatus* Riccob. | Xoconostle | IT463 |

**Appendix 1**. Continuation…

| **No.** | **Family** | **Species** | **Common name** | **Voucher specimen** |
| --- | --- | --- | --- | --- |
| 39 | Caricaceae | *Carica papaya* L. | Papaya | Photo record |
| 40 | Chenopodiaceae | *Chenopodium ambrosioides* L. | Epazote | YA574, YA694, YA727 |
| 41 | Chenopodiaceae | *Chenopodium berlandieri* Moq. | Huauzontle | YE375 |
| 42 | Convolvulaceae | *Ipomoea batatas* (L.) Lam. | Camote | Photo record |
| 43 | Cucurbitaceae | *Apodanthera aspera* Cogn. | Pisto | RL1093 |
| 44 | Cucurbitaceae | *Cucumis melo* L. | Melón | Photo record |
| 45 | Cucurbitaceae | *Cucurbita argyrosperma* K. Koch. subsp. *argyrosperma* | Calabaza | RL1109, RL1112, RL1113 |
| 46 | Cucurbitaceae | *Cucurbita ficifolia* Bouché | Chilacayota | Photo record |
| 47 | Cucurbitaceae | *Cucurbita mostacha* (Duch. ex Lam.) Duch. ex Poiret | Calabaza tamala | RL1092, RL1095, RL1108 |
| 48 | Cucurbitaceae | *Cucurbita pepo* L. | calabaza | SR184 |
| 49 | Cucurbitaceae | *Sechium edule* (Jacq.) Sw. | Chayote | ER215 |
| 50 | Dioscoreaceae | *Dioscorea* sp. | Chichicamotl | Photo record |
| 51 | Ebenaceae | *Diospyros digyna* Jacq. | Zapote negro | Photo record |
| 52 | Euphorbiaceae | *Jatropha neopauciflora* Pax | Piñon de la Mixteca | YA716, YA757 |
| 53 | Euphorbiaceae | *Manihot esculenta* Crantz | Yuca | Photo record |
| 54 | Fabaceae | *Crotalaria pumila* Ortega | Chepil | YA584, SR103 |
| 55 | Fabaceae | *Erythrina americana* Miller | Pipe | YA641, YA748, SR458 |
| 56 | Fabaceae | *Inga vera* Willd. | Cuajinicuil | Photo record |
| 57 | Fabaceae | *Leucaena esculenta* (Mociño & Sessé ex DC) Benth. | Huaje rojo | SR1216 |
| 58 | Fabaceae | *Leucaena leucocephala* (Lam.) de Wit. | Huajes | YA582, YA616 |
| 59 | Fabaceae | *Pachyrhizus erosus* (L.) Urb. | Jicama | Photo record |
| 60 | Fabaceae | *Phaseolus coccineus* L. | Ejote | YA589, YA634, YA735 |
| 61 | Fabaceae | *Phaseolus* sp. | Flor de frijol | Photo record |
| 62 | Fabaceae | *Phaseolus vulgaris* L. | Frijol | SR9 |
| 63 | Fabaceae | *Pisum sativum* L. | Chícharo | Photo record |
| 64 | Fabaceae | *Pithecellobium dulce* (Roxb.) Benth. | Guamuchil | Photo record |
| 65 | Fabaceae | *Tamarindus indica* L. | Tamarindo | Photo record |
| 66 | Fabaceae | *Vicia faba* L. | Haba | Photo record |
| 67 | Lamiaceae | *Menta piperita* L. | Hierba buena | YA576, YA624, YA744 |
| 68 | Lamiaceae | *Origanum majorana* L. | Mejorana | YA672, SR206 |
| 69 | Lamiaceae | *Clinopodium mexicanum* (Benth.) Govaerts | Hierba del aire | SR1280 |
| 70 | Lamiaceae | *Thymus vulgaris* L. | Tomillo fresco | YA614, YA668, YA670 |
| 71 | Lauraceae | *Litsea glaucescens* Kunth | Laurel | YA669, YA676, YA746 |
| 72 | Lauraceae | *Litsea neesiana* (S. Schauer) Hemsl. | Laurel | YA671 |
| 73 | Lauraceae | *Persea americana* Miller | Aguacate | YA611, YA630, YA688 |
| 74 | Lythraceae | *Punica granatum* L. | Granada | EPN142, SR43 |
| 75 | Malpighiaceae | *Byrsonima crassifolia* (L.) Kunth | Nanche | AVB126, AVB192, AVB499 |
| 76 | Malpighiaceae | *Malpighia* sp. | Nanche rojo | Photo record |
| 77 | Malvaceae | *Abelmoschus esculentus* (L.) Moench | Angú | YA590 |
| 78 | Malvaceae | *Ceiba aesculifolia* subsp. *parvifolia* (Rose) P.E. Gibbs & Semir | Pochote | YA651, EPN61, YE244 |
| 79 | Musaceae | *Musa paradisiaca* L. | Plátano | Photo record |
| 80 | Myrtaceae | *Psidium guajava* Griseb. | Guayaba | EPN122 |
| 81 | Nolineaceae | *Dasylirion serratifolium* Lem. | Manitas | YA591, YA627, YA704 |
| 82 | Oxalidaceae | *Averrhoa carambola* L. | Carambolo | Photo record |
| 83 | Oxalidaceae | *Oxalis tuberosa* Molina | Papa extranjera | Photo record |
| 84 | Passifloraceae | *Passiflora edulis* Sims | Granada china | LS180 |
| 85 | Passifloraceae | *Passiflora ligularis* Juss. | Maracuya | Photo record |
| 86 | Phytolaccaceae | *Phytolacca icosandra* L. | Quelite | YA720, LS309 |

**Appendix 1**. Finish..

| **No.** | **Family** | **Species** | **Common name** | **Voucher specimen** |
| --- | --- | --- | --- | --- |
| 87 | Pinaceae | *Pinus cembroides* Zucc. | Piñon | Photo record |
| 88 | Piperaceae | *Peperomia peltilimba* C. DC. | Tequelite | YA577 |
| 89 | Piperaceae | *Piper auritum* Kunth | Hierba santa | SR418 |
| 90 | Poaceae | *Triticum aestivum* L. | Trigo | SR172 |
| 91 | Poaceae | *Zea mays* L. | Maíz | SR174 |
| 92 | Polygonaceae | *Rumex crispus* L. | Lengua de vaca | YA573, YA687, YA723 |
| 93 | Portulacaceae | *Portulaca oleracea* L. | Verdolaga | LS397, IT152 |
| 94 | Rosaceae | *Crataegus pubescens* Moc. & Sessé ex DC. | Tejocote | SR1424 |
| 95 | Rosaceae | *Eriobotrya japonica* (Thunb.) Lindl. | Níspero | SR50 |
| 96 | Rosaceae | *Fragaria × ananassa* Rozier Duchesne ex Rozier | Fresa | Photo record |
| 97 | Rosaceae | *Malus pumila* Mill. | Manzana | SR227 |
| 98 | Rosaceae | *Prunus persica* (L.) Batsch | Durazno | SR226 |
| 99 | Rosaceae | *Prunus serotina* Ehrh. | Capulín | SR1412 |
| 100 | Rosaceae | *Pyrus communis* L. | Pera | Photo record |
| 101 | Rubiaceae | *Coffea arabica* L. | Cafe | Photo record |
| 102 | Rutaceae | *Citrus grandis*  (L.) Osbeck | Lima | Photo record |
| 103 | Rutaceae | *Citrus medica* L | Limón | EPN123 |
| 104 | Sapotaceae | *Couepia poliandra* (Kunth) Rose | Zapote de niño | Photo record |
| 105 | Sapotaceae | *Manilkara zapota* (L.) P. Royen | Chicozapote | EPN124 |
| 106 | Sapotaceae | *Sideroxylon palmeri* (Rose) T.D. Penn. | Tempesquistle | YA642, YA663, YA706 |
| 107 | Solanaceae | *Capsicum annuum* L. | Chile miahuateco | YA734 |
| 108 | Solanaceae | *Capsicum annuum* var*. annuum* L. | Chile de monte | Photo record |
| 109 | Solanaceae | *Capsicum annuum* var. *aviculare* L. | Chile adobo | Photo record |
| 110 | Solanaceae | *Capsicum pubescens* Ruiz & Pav. | Chile canario | ER181 |
| 111 | Solanaceae | *Cestrum nocturmun* L. | Huele de noche | YA656, YA678 |
| 112 | Solanaceae | *Physalis philadelphica* Lam. | Tomate verde | EPN62, SR26 |
| 113 | Solanaceae | *Sp. 1* | Tomatillo | YA702 |
| 114 | Solanaceae | *Solanum lycopersicum* L. | Jitomate | IT391 |
| 115 | Solanaceae | *Solanum nigrescens* M. Martens & Galeotti | Hierba mora | YA615, YA655, YA689 |
| 116 | Solanaceae | *Solanum* sp. | Quelite | Photo record |
| 117 | Solanaceae | *Solanum tuberosum* L. | Papa | Photo record |
| 118 | Solanaceae | *Solanum melongena* L. | Berenjena | Photo record |
| 119 | Solanaceae | *Witheringia solanacea* L'Hér. | Hierba mora | YA679 |
| 120 | Verbenaceae | *Lippia graveolens* Cav. | Oregano | YA575, EPN60 |
| 121 | Vitaceae | *Vitis* sp. | Uva silvestre | Photo record |
| 122 | Zingiberaceae | *Renealmia alpinia* (Rottb.) Maas | Guasmole | YA643 |

**Appendix 2** Data used in Partial Canonical Ordination Analysis. Asterisks show raw data not used in the analysis.Some variables were converted to words and words to numbers, such as: *Origin*: native=2, no native=1; *Ecological Status*: si: wild=1, do:domesticated=2, ar:weed or ruderal=3; *Management*: Cu:cultivated=5, Fo: fomented or promoted=3, Pr:protected=4, To:tolerated or cultivated=2, Re:recollected or gathered=1; and in *Management System*: Vn:Natural vegetation=1, Vs:secondary vegetation=2, Sa: Agroforestal System=3, H:homegardens=4, Si: Intensive system=5.

| **Family** | ***Species name*** | **Ecological Marix (Matrix X)** | | | | | | | | **Socioeconomic Matrix (Matrix W)** | | | | | | | | **Management Matrix (Response Matrix Y)** | | | | | | | |
| --- | --- | --- | --- | --- | --- | --- | --- | --- | --- | --- | --- | --- | --- | --- | --- | --- | --- | --- | --- | --- | --- | --- | --- | --- | --- |
|  |  | **No. Plots** | **% Plot** | **No. types Veg** | **% Veg** | **Months present** | **% Month present** | **Part Used Index** | **Life Cycle** | **No. Market** | **% Market** | **Markt Stall** | **Price min** | **Price max** | **Price Avera.** | **Vol. Sold** | **Inter-chan-ged** | **Origin** | **Ecolog. Status** | **Manag-ment.** | **Manag-ement System** | **Cat. Ecolog Status** | **Cat Manag** | **Cat. Manag. Sys** | **No. Uses** |
| Asparagaceae | *Agave potatorum* | 14 | 14.3 | 2 | 0.75 | 2 | 16.67 | 0.75 | 1 | 3 | 50 | 1 | 12.2 | 17.5 | 15.44 | 2.7 | 2 | 2 | do | Cu | SiHSa | 1 | 5 | 3 | 12 |
| Asparagaceae | *Agave sp.1* | 1 | 1.02 | 1 | 0.13 | 5 | 41.67 | 0.75 | 1 | 3 | 50 | 5 | 6.72 | 25.9 | 11.81 | 131.2 | 2 | 2 | ar | FoReTo | SaVs | 1 | 4 | 1 | 1 |
| Asparagaceae | *Agave sp.2* | 1 | 1.02 | 1 | 0.13 | 4 | 33.33 | 0.75 | 1 | 4 | 67 | 5 | 8.07 | 35.5 | 15.47 | 46.8 | 2 | 2 | ar | Cu | HSaVs | 1 | 4 | 1 | 1 |
| Asparagaceae | *Yucca periculosa* | 3 | 3.06 | 3 | 0.5 | 1 | 8.33 | 0.75 | 1 | 3 | 50 | 1.33 | 9.3 | 24 | 16.56 | 7.5 | 2 | 2 | dosi | Cu | HSaVs | 1 | 4 | 2 | 7 |
| Alliaceae | *Allium cepa* | 1 | 1.02 | 6 | 0.13 | 2 | 16.67 | 1.5 | 2 | 4 | 67 | 2 | 11.1 | 11.1 | 11.10 | 20.0 | 2 | 1 | si | Cu | SiHSaVn | 1 | 1 | 2 | 2 |
| Alliaceae | *Allium sativum* | 1 | 1.02 | 1 | 0.13 | 2 | 16.67 | 1.5 | 2 | 1 | 17 | 2 | 14.3 | 14.3 | 14.30 | 5.0 | 2 | 1 | do | Cu | HSa | 1 | 1 | 2 | 3 |
| Amaranthaceae | *Amaranthus hybridus* | 7 | 7.14 | 1 | 0.38 | 7 | 58.33 | 3 | 2 | 6 | 100 | 6 | 5 | 25.2 | 17.38 | 300.1 | 2 | 2 | si | Cu | HSaVs | 2 | 4 | 4 | 3 |
| Anacardiaceae | *Cyrtocarpa procera* | 3 | 3.06 | 3 | 0.25 | 2 | 16.67 | 1.5 | 1 | 4 | 67 | 2 | 10.1 | 22.2 | 18.65 | 71.6 | 2 | 2 | do | Cu | Sa | 1 | 3 | 3 | 8 |
| Anacardiaceae | *Spondias mombin* | 1 | 1.02 | 1 | 0.13 | 3 | 25 | 1.5 | 1 | 1 | 17 | 1 | 13 | 13 | 12.50 | 5.0 | 2 | 2 | do | Cu | H | 2 | 2 | 2 | 4 |
| Anacardiaceae | *Spondias purpurea* | 1 | 1.02 | 2 | 0.13 | 2 | 16.67 | 1.5 | 1 | 3 | 50 | 4.5 | 10 | 10 | 10.00 | 70.0 | 1 | 2 | do | Cu | H | 1 | 3 | 3 | 1 |
| Annonaceae | *Annona cherimola* | 5 | 5.1 | 1 | 0.38 | 4 | 33.33 | 1.5 | 1 | 2 | 33 | 1 | 11.4 | 23.8 | 17.59 | 22.6 | 2 | 1 | do | Cu | H | 2 | 1 | 3 | 6 |
| Annonaceae | *Annona reticulata* | 1 | 1.02 | 2 | 0.13 | 6 | 50 | 1.5 | 1 | 3 | 50 | 2 | 11.4 | 12.5 | 12.02 | 16.5 | 2 | 1 | do | Cu | H | 1 | 2 | 2 | 1 |
| Apiaceae | *Coriandrum sativum* | 1 | 1.02 | 1 | 0.13 | 3 | 25 | 3 | 2 | 6 | 100 | 5 | 9.8 | 50.5 | 25.18 | 40.7 | 2 | 1 | do | Cu | H | 1 | 1 | 1 | 2 |
| Araceae | *Spathiphyllum cochlearispathum* | 1 | 1.02 | 3 | 0.13 | 1 | 8.33 | 0.75 | 2 | 1 | 17 | 1 | 30 | 30 | 30.30 | 0.2 | 1 | 2 | do | Cu | H | 1 | 1 | 3 | 1 |
| Arecaceae | *Chamaedorea tepejilote* | 4 | 4.08 | 1 | 0.25 | 2 | 16.67 | 1.5 | 1 | 5 | 83 | 5 | 8.62 | 19 | 14.36 | 661.9 | 2 | 1 | do | Cu | Si | 1 | 1 | 4 | 1 |
| Asteraceae | *Porophyllum linaria* | 8 | 8.16 | 3 | 0.63 | 3 | 25 | 3 | 2 | 6 | 100 | 5.8 | 18 | 50 | 27.05 | 2.0 | 2 | 2 | do | Cu | SaVs | 2 | 4 | 5 | 3 |
| Asteraceae | *Porophyllum macrocephalum* | 3 | 3.06 | 2 | 0.38 | 3 | 25 | 3 | 2 | 6 | 100 | 6 | 18.7 | 65 | 30.98 | 28.5 | 2 | 2 | do | Cu | HSa | 2 | 5 | 3 | 3 |
| Asteraceae | *Sonchus oleraceus* | 1 | 1.02 | 2 | 0.13 | 2 | 16.67 | 3 | 2 | 1 | 17 | 1 | 6.3 | 6.3 | 6.26 | 4.8 | 2 | 1 | do | Cu | HSa | 1 | 1 | 3 | 1 |
| Bignoniaceae | *Parmentiera edulis* | 1 | 1.02 | 1 | 0.13 | 3 | 25 | 1.5 | 2 | 2 | 33 | 1.5 | 9.4 | 9.4 | 16.03 | 25.0 | 2 | 1 | si | CuTo | HSa | 2 | 1 | 2 | 6 |
| Bixaceae | *Bixa orellana* | 1 | 1.02 | 1 | 0.13 | 3 | 25 | 1 | 1 | 2 | 33 | 1 | 90.9 | 125 | 107.95 | 1.2 | 1 | 1 | ar | Fo | HSaVs | 1 | 1 | 1 | 1 |

**Appendix 2** Continuation…..

| **Family** | ***Species name*** | **Ecological Marix (Matrix X)** | | | | | | | | **Socioeconomic Matrix (Matrix W)** | | | | | | | | **Management Matrix (Response Matrix Y)** | | | | | | | |
| --- | --- | --- | --- | --- | --- | --- | --- | --- | --- | --- | --- | --- | --- | --- | --- | --- | --- | --- | --- | --- | --- | --- | --- | --- | --- |
|  |  | **No. Plots** | **% Plot** | **No. types Veg** | **% Veg** | **Months present** | **% Month present** | **Part Used Index** | **Life Cycle** | **No. Market** | **% Market** | **Markt Stall** | **Price min** | **Price max** | **Price Avera.** | **Vol. Sold** | **Interch-anged.** | **Origin** | **Ecolog. Status** | **Manag-ment.** | **Manag-ement System** | **Cat. Ecolog Status** | **Cat Manag** | **Cat. Manag. Sys** | **No. Uses** |
| Malvaceae | *Ceiba aesculifolia* subsp. *parvifolia* | 11 | 11.2 | 1 | 0.38 | 4 | 33.33 | 1 | 1 | 3 | 50 | 4.667 | 22.7 | 116 | 43.77 | 231.6 | 2 | 2 | do | Cu | HVs | 1 | 3 | 4 | 10 |
| Brassicaceae | *Brassica campestris* | 3 | 3.06 | 1 | 0.25 | 7 | 58.33 | 3 | 2 | 6 | 100 | 4 | 6.1 | 15 | 10.12 | 71.2 | 2 | 1 | doar | Cu | SiSa | 1 | 3 | 2 | 3 |
| Brassicaceae | *Brassica oleracea* var. *viridis* | 1 | 1.02 | 1 | 0.13 | 1 | 8.33 | 1.5 | 2 | 1 | 17 | 1 | 20 | 22.2 | 21.11 | 1.1 | 1 | 1 | do | Cu | SiHSa | 1 | 1 | 1 | 1 |
| Brassicaceae | *Raphanus sativus* | 1 | 1.02 | 1 | 0.13 | 4 | 33.33 | 1.5 | 2 | 6 | 100 | 4 | 12.5 | 12.5 | 12.50 | 60.0 | 2 | 1 | do | Cu | SiHSa | 2 | 1 | 2 | 2 |
| Brassicaceae | *Rorippa nasturtium-aquaticum* | 1 | 1.02 | 1 | 0.13 | 5 | 41.67 | 3 | 2 | 6 | 100 | 5 | 14 | 28 | 17.90 | 35.4 | 2 | 1 | do | Cu | SiH | 1 | 2 | 2 | 2 |
| Cactaceae | *Acanthocereus subinermis* | 1 | 1.02 | 1 | 0.13 | 2 | 16.67 | 1 | 1 | 2 | 33 | 2 | 16.7 | 22.7 | 19.70 | 3.4 | 2 | 1 | do | Cu | H | 1 | 1 | 2 | 2 |
| Cactaceae | *Escontria chiotilla* | 11 | 11.2 | 1 | 0.25 | 5 | 41.67 | 1.5 | 1 | 4 | 67 | 5 | 11.4 | 28.4 | 14.59 | 127.4 | 2 | 2 | ar | Cu | Sa | 2 | 5 | 4 | 7 |
| Cactaceae | *Ferocactus latispinus* | 11 | 11.2 | 2 | 0.25 | 2 | 16.67 | 3 | 1 | 1 | 17 | 1 | 111 | 111 | 111.11 | 1.1 | 1 | 2 | do | Cu | SiH | 1 | 4 | 4 | 4 |
| Cactaceae | *Hylocereus undatus* | 1 | 1.02 | 1 | 0.13 | 5 | 41.67 | 1.5 | 1 | 5 | 83 | 2 | 7 | 8 | 7.67 | 11.0 | 1 | 2 | do | Cu | H | 2 | 2 | 2 | 3 |
| Cactaceae | *Myrtillocactus geometrizans* | 8 | 8.16 | 3 | 0.38 | 1 | 8.33 | 1.5 | 1 | 1 | 17 | 1 | 40 | 79 | 59.13 | 4.7 | 1 | 2 | do | Cu | H | 1 | 3 | 4 | 9 |
| Cactaceae | *Neobuxbaumia tetetzo* | 4 | 4.08 | 1 | 0.38 | 3 | 25 | 0.75 | 1 | 2 | 33 | 1 | 36 | 51 | 43.33 | 27.8 | 1 | 2 | do | Cu | H | 1 | 3 | 2 | 6 |
| Cactaceae | *Opuntia auberi* | 1 | 1.02 | 1 | 0.13 | 4 | 33.33 | 0.75 | 1 | 1 | 17 | 1 | 11 | 12 | 11.77 | 4.3 | 1 | 1 | doar | Cu | HSa | 1 | 2 | 2 | 4 |
| Cactaceae | *Opuntia sp.* | 1 | 1.02 | 1 | 0.13 | 4 | 33.33 | 1 | 1 | 3 | 50 | 1.5 | 11.9 | 15 | 13.46 | 7.5 | 1 | 1 | dosi | CuPr | HSaVn | 1 | 2 | 2 | 3 |
| Cactaceae | *Pachycereus weberi* | 11 | 11.2 | 1 | 0.5 | 2 | 16.67 | 1 | 1 | 1 | 17 | 1 | 122 | 188 | 154.97 | 2.2 | 1 | 2 | ar | CuRe | HVs | 1 | 2 | 2 | 7 |
| Cactaceae | *Stenocereus pruinosus* | 5 | 5.1 | 2 | 0.38 | 2 | 16.67 | 1.5 | 1 | 3 | 50 | 9 | 14 | 21 | 17.71 | 470.0 | 2 | 2 | dosi | Cu | SiSa | 2 | 4 | 5 | 7 |
| Cactaceae | *Stenocereus stellatus* | 10 | 10.2 | 1 | 0.38 | 3 | 25 | 1.5 | 1 | 4 | 67 | 5 | 3.21 | 20 | 11.73 | 410.0 | 2 | 2 | arsi | Cu | HSaVs | 2 | 4 | 4 | 8 |
| Caricaceae | *Carica papaya* | 4 | 4.08 | 2 | 0.38 | 3 | 25 | 1.5 | 1 | 4 | 67 | 1 | 5 | 10 | 7.50 | 2.0 | 2 | 1 | do | CuFo | HSa | 1 | 1 | 3 | 4 |
| Chenopodiaceae | *Chenopodium ambrosioides* | 1 | 1.02 | 1 | 0.13 | 9 | 75 | 3 | 2 | 6 | 100 | 3 | 10 | 18.2 | 14.76 | 24.6 | 2 | 2 | do | Cu | H | 2 | 4 | 3 | 4 |
| Chenopodiaceae | *Chenopodium berlandieri* | 1 | 1.02 | 3 | 0.13 | 3 | 25 | 0.75 | 2 | 1 | 17 | 1 | 11 | 11 | 3.66 | 4.6 | 1 | 2 | si | Cu | HSa | 1 | 2 | 2 | 2 |
| Convolvulaceae | *Ipomoea batatas* | 1 | 1.02 | 4 | 0.13 | 3 | 25 | 1.5 | 2 | 4 | 67 | 1 | 5 | 17.5 | 10.61 | 12.0 | 2 | 1 | do | CuFo | HSa | 2 | 1 | 2 | 1 |
| Cucurbitaceae | *Apodanthera aspera* | 1 | 1.02 | 1 | 0.13 | 2 | 16.67 | 1 | 1 | 1 | 17 | 1 | 100 | 417 | 308.50 | 2.2 | 1 | 2 | arsi | Cu | HSa | 2 | 3 | 2 | 1 |

**Appendix 2** Continuation…..

| **Family** | ***Species name*** | **Ecological Marix (Matrix X)** | | | | | | | | **Socioeconomic Matrix (Matrix W)** | | | | | | | | **Management Matrix (Response Matrix Y)** | | | | | | | |
| --- | --- | --- | --- | --- | --- | --- | --- | --- | --- | --- | --- | --- | --- | --- | --- | --- | --- | --- | --- | --- | --- | --- | --- | --- | --- |
|  |  | **No. Plots** | **% Plot** | **No. types Veg** | **% Veg** | **Months present** | **% Month present** | **Part Used Index** | **Life Cycle** | **No. Market** | **% Market** | **Markt Stall** | **Price min** | **Price max** | **Price Avera.** | **Vol. Sold** | **Interch-anged.** | **Origin** | **Ecolog. Status** | **Manag-ment.** | **Manag-ement System** | **Cat. Ecolog Status** | **Cat Manag** | **Cat. Manag. Sys** | **No. Uses** |
| Cucurbitaceae | *Cucumis melo* | 1 | 1.02 | 1 | 0.13 | 3 | 25 | 1.5 | 2 | 3 | 50 | 1 | 9 | 12.5 | 10.67 | 50.0 | 2 | 1 | si | CuReTo | Vn | 1 | 1 | 1 | 1 |
| Cucurbitaceae | *Cucurbita ficifolia* | 1 | 1.02 | 2 | 0.13 | 4 | 33.33 | 1.5 | 2 | 3 | 50 | 1.5 | 5 | 39.2 | 13.07 | 50.0 | 1 | 1 | do | Cu | SiH | 1 | 1 | 1 | 2 |
| Cucurbitaceae | *Cucurbita mostacha* | 1 | 1.02 | 1 | 0.13 | 2 | 16.67 | 1 | 2 | 1 | 17 | 1 | 33.3 | 33.3 | 11.11 | 7.0 | 1 | 2 | do | Cu | Sa | 1 | 1 | 2 | 3 |
| Cucurbitaceae | *Cucurbita pepo* | 4 | 4.08 | 1 | 0.25 | 3 | 25 | 0.75 | 2 | 6 | 100 | 4.5 | 11.1 | 33.3 | 11.11 | 90.0 | 2 | 2 | do | Cu | SiHSa | 1 | 1 | 3 | 4 |
| Cucurbitaceae | *Sechium edule* | 1 | 1.02 | 1 | 0.13 | 7 | 58.33 | 3 | 2 | 6 | 100 | 5 | 6.06 | 11.1 | 8.50 | 100.0 | 2 | 2 | do | CuPrTo | SiHSaVs | 2 | 1 | 2 | 1 |
| Dioscoreaceae | *Dioscorea sp* | 1 | 1.02 | 3 | 0.13 | 2 | 16.67 | 1 | 1 | 1 | 17 | 1 | 33.3 | 33.3 | 11.11 | 7.0 | 1 | 2 | ar | Cu | HSa | 1 | 3 | 1 | 2 |
| Euphorbiaceae | *Jatropha neopauciflora* | 1 | 1.02 | 1 | 0.13 | 3 | 25 | 1 | 1 | 2 | 33 | 1.5 | 100 | 129 | 80.51 | 5.7 | 2 | 1 | si | CuFoReTo | SaVn | 1 | 3 | 1 | 2 |
| Euphorbiaceae | *Manihot esculenta* | 1 | 1.02 | 1 | 0.13 | 2 | 16.67 | 1.5 | 1 | 1 | 17 | 1 | 5 | 23 | 7.72 | 5.4 | 1 | 1 | arsi | FoReTo | HSaVs | 1 | 1 | 2 | 1 |
| Fabaceae | *Crotalaria pumila* | 1 | 1.02 | 1 | 0.13 | 3 | 25 | 3 | 2 | 1 | 17 | 1 | 5.71 | 5.71 | 1.90 | 3.5 | 1 | 2 | arsi | CuFoPrReTo | SiHVn | 2 | 2 | 3 | 4 |
| Fabaceae | *Erythrina americana* | 4 | 4.08 | 1 | 0.38 | 3 | 25 | 0.75 | 1 | 2 | 33 | 2 | 10.2 | 22.5 | 12.17 | 6.6 | 1 | 1 | si | CuPrReTo | HSaVsVn | 1 | 1 | 3 | 8 |
| Fabaceae | *Inga vera* | 1 | 1.02 | 2 | 0.13 | 3 | 25 | 1.5 | 1 | 3 | 50 | 1.5 | 10 | 15 | 8.33 | 7.0 | 1 | 2 | dosi | CuPrReTo | SiHSaVsVn | 1 | 1 | 4 | 1 |
| Fabaceae | *Leucaena esculenta* | 1 | 1.02 | 1 | 0.13 | 3 | 25 | 1.5 | 1 | 6 | 100 | 3.333 | 15 | 24 | 18.73 | 70.0 | 2 | 2 | si | CuFoRe | HVsVn | 2 | 2 | 5 | 8 |
| Fabaceae | *Leucaena leucocephala* | 1 | 1.02 | 1 | 0.13 | 3 | 25 | 1.5 | 1 | 3 | 50 | 2.5 | 18.5 | 23.5 | 20.68 | 2.5 | 2 | 2 | si | CuFoTo | HSaVs | 2 | 4 | 4 | 7 |
| Fabaceae | *Pachyrhizus erosus* | 1 | 1.02 | 1 | 0.13 | 2 | 16.67 | 1.5 | 1 | 3 | 50 | 1 | 3.13 | 17 | 3.14 | 5.3 | 1 | 1 | si | CuFoPrReTo | HSaVn | 2 | 1 | 2 | 2 |
| Fabaceae | *Phaseolus coccineus* | 2 | 2.04 | 1 | 0.25 | 2 | 16.67 | 1.5 | 2 | 5 | 83 | 3.5 | 12.5 | 13 | 12.80 | 50.0 | 2 | 2 | dosi | Cu | SiHSa | 2 | 1 | 3 | 3 |
| Fabaceae | *Phaseolus* sp. | 1 | 1.02 | 3 | 0.13 | 2 | 16.67 | 0.75 | 2 | 2 | 33 | 2.5 | 4 | 16 | 8.79 | 9.3 | 1 | 2 | dosi | CuReTo | HSaVsVn | 2 | 1 | 2 | 1 |
| Fabaceae | *Phaseolus vulgaris* | 2 | 2.04 | 2 | 0.25 | 4 | 33.33 | 1 | 2 | 5 | 83 | 2 | 11 | 15 | 13.43 | 151.0 | 1 | 2 | si | CuReTo | HSaVsVn | 2 | 1 | 3 | 2 |
| Fabaceae | *Pithecellobium dulce* | 2 | 2.04 | 2 | 0.25 | 1 | 8.33 | 1.5 | 1 | 1 | 17 | 1 | 15 | 15 | 15.00 | 5.0 | 1 | 2 | si | FoReTo | HSaVn | 2 | 3 | 4 | 12 |
| Fabaceae | *Tamarindus indica* | 1 | 1.02 | 1 | 0.13 | 3 | 25 | 1.5 | 1 | 2 | 33 | 1 | 7.8 | 10 | 8.89 | 22.0 | 1 | 1 | arsi | CuFoPrRe | SiHSaVsVn | 1 | 3 | 4 | 5 |
| Fabaceae | *Vicia faba* | 1 | 1.02 | 2 | 0.13 | 3 | 25 | 1.5 | 2 | 4 | 67 | 4.5 | 14 | 14 | 14.29 | 2.5 | 2 | 1 | dosi | Cu | HSaVs | 1 | 1 | 3 | 1 |
| Lamiaceae | *Menta* x *piperita* | 1 | 1.02 | 3 | 0.13 | 4 | 33.33 | 3 | 2 | 6 | 100 | 2 | 18 | 36 | 26.10 | 6.7 | 2 | 1 | si | ToRe | SaVn | 1 | 1 | 1 | 2 |
| Lamiaceae | *Origanum majorana* | 1 | 1.02 | 2 | 0.13 | 2 | 16.67 | 3 | 2 | 2 | 33 | 1.5 | 283 | 333 | 308.18 | 0.4 | 1 | 1 | dosi | CuFoPrReTo | HSaVsVn | 1 | 1 | 1 | 2 |
| Lamiaceae | *Clinopodium mexicanum* | 1 | 1.02 | 1 | 0.13 | 2 | 16.67 | 3 | 2 | 4 | 67 | 4.5 | 13 | 14 | 13.46 | 89.7 | 2 | 2 | si | FoReTo | Vn | 1 | 3 | 2 | 2 |
| Lamiaceae | *Thymus vulgaris* | 1 | 1.02 | 1 | 0.13 | 2 | 16.67 | 3 | 2 | 3 | 50 | 2.33 | 57 | 188 | 91.36 | 1.8 | 2 | 1 | si | CuReTo | SaVn | 1 | 1 | 1 | 2 |
| Lauraceae | *Litsea glaucescens* | 5 | 5.1 | 3 | 0.5 | 3 | 25 | 2 | 1 | 4 | 67 | 2.5 | 6.4 | 118 | 47.61 | 18.7 | 2 | 2 | doar | PrCu | SiHVs | 1 | 3 | 3 | 1 |

**Appendix 2** Continuation…..

| **Family** | ***Species name*** | **Ecological Marix (Matrix X)** | | | | | | | | **Socioeconomic Matrix (Matrix W)** | | | | | | | | **Management Matrix (Response Matrix Y)** | | | | | | | |
| --- | --- | --- | --- | --- | --- | --- | --- | --- | --- | --- | --- | --- | --- | --- | --- | --- | --- | --- | --- | --- | --- | --- | --- | --- | --- |
|  |  | **No. Plots** | **% Plot** | **No. types Veg** | **% Veg** | **Months present** | **% Month present** | **Part Used Index** | **Life Cycle** | **No. Market** | **% Market** | **Markt Stall** | **Price min** | **Price max** | **Price Avera.** | **Vol. Sold** | **Interch-anged.** | **Origin** | **Ecolog. Status** | **Manag-ment.** | **Manag-ement System** | **Cat. Ecolog Status** | **Cat Manag** | **Cat. Manag. Sys** | **No. Uses** |
| Lauraceae | *Litsea neesiana* | 1 | 1.02 | 1 | 0.13 | 1 | 8.33 | 2 | 1 | 1 | 17 | 1 | 41 | 41 | 41.08 | 2.4 | 1 | 2 | si | CuReTo | HSaVsVn | 1 | 3 | 1 | 1 |
| Lauraceae | *Persea americana* | 7 | 7.14 | 1 | 0.38 | 3 | 25 | 2 | 1 | 6 | 100 | 4 | 3.2 | 13 | 7.95 | 36.7 | 2 | 2 | dosi | CuFoPrReTo | HSaVsVn | 1 | 1 | 3 | 4 |
| Malpighiaceae | *Byrsonima crassifolia* | 1 | 1.02 | 1 | 0.13 | 3 | 25 | 1.5 | 1 | 2 | 33 | 1 | 22.7 | 25 | 23.86 | 20.0 | 1 | 2 | si | CuRe | HSaVsVn | 2 | 3 | 2 | 4 |
| Malpighiaceae | *Malpighia* sp. | 1 | 1.02 | 6 | 0.13 | 1 | 8.33 | 1.5 | 1 | 1 | 17 | 1 | 27 | 27 | 27.03 | 0.7 | 1 | 1 | dosi | CuPrReTo | HSaVsVn | 1 | 1 | 2 | 1 |
| Malvaceae | *Abelmoschus esculentus* | 1 | 1.02 | 2 | 0.13 | 2 | 16.67 | 0.75 | 2 | 2 | 33 | 2 | 12.5 | 15.6 | 14.06 | 9.6 | 1 | 1 | arsi | FoPrReTo | SiHSaVs | 1 | 1 | 1 | 2 |
| Nolineaceae | *Dasylirion serratifolium* | 8 | 8.16 | 1 | 0.63 | 6 | 50 | 0.75 | 2 | 3 | 50 | 3.5 | 15.6 | 42.6 | 23.74 | 32.1 | 2 | 2 | do | Cu | HSaVs | 1 | 3 | 1 | 2 |
| Oxalidaceae | *Averrhoa carambola* | 1 | 1.02 | 1 | 0.13 | 2 | 16.67 | 1.5 | 2 | 2 | 33 | 1.5 | 9.09 | 10 | 9.55 | 51.0 | 2 | 1 | do | Cu | SiHSa | 1 | 1 | 1 | 1 |
| Oxalidaceae | *Oxalis tuberosa* | 1 | 1.02 | 1 | 0.13 | 1 | 8.33 | 1.5 | 1 | 2 | 33 | 6 | 3.54 | 7.6 | 6.41 | 26.0 | 2 | 1 | si | FoReTo | Vn | 1 | 1 | 2 | 1 |
| Phytolaccaceae | *Phytolacca icosandra* | 2 | 2.04 | 2 | 0.25 | 2 | 16.67 | 3 | 1 | 1 | 17 | 1 | 7.27 | 7.27 | 7.27 | 3.1 | 1 | 2 | si | FoPrReTo | Vn | 2 | 3 | 3 | 1 |
| Pinaceae | *Pinus cembroides* | 1 | 1.02 | 1 | 0.13 | 3 | 25 | 1 | 1 | 1 | 17 | 1 | 45 | 45 | 45.45 | 20.0 | 1 | 2 | si | FoPrReTo | Vn | 1 | 1 | 2 | 1 |
| Piperaceae | *Peperomia peltilimba* | 1 | 1.02 | 2 | 0.13 | 2 | 16.67 | 3 | 1 | 1 | 17 | 1 | 40 | 40 | 40.00 | 0.5 | 1 | 2 | ar | ToRe | Sa | 1 | 3 | 3 | 1 |
| Piperaceae | *Piper auritum Kunth* | 1 | 1.02 | 1 | 0.13 | 1 | 8.33 | 3 | 1 | 2 | 33 | 2 | 13 | 15 | 14.70 | 2.0 | 1 | 1 | si | Cu | SaVn | 1 | 1 | 2 | 5 |
| Poaceae | *Triticum aestivum* | 1 | 1.02 | 1 | 0.13 | 2 | 16.67 | 1 | 1 | 1 | 17 | 1 | 12 | 12 | 12.00 | 15.0 | 1 | 1 | arsi | CuReTo | SaVs | 1 | 1 | 1 | 4 |
| Polygonaceae | *Rumex crispus* | 1 | 1.02 | 1 | 0.13 | 3 | 25 | 3 | 1 | 2 | 33 | 2.5 | 12 | 17 | 14.28 | 10.6 | 1 | 1 | ar | Re | HSa | 2 | 1 | 3 | 3 |
| Portulacaceae | *Portulaca oleracea* | 3 | 3.06 | 2 | 0.25 | 1 | 8.33 | 3 | 2 | 2 | 33 | 1 | 6.9 | 9 | 8.02 | 10.5 | 1 | 2 | dosi | Cu | HSa | 1 | 3 | 3 | 4 |
| Rosaceae | *Crataegus pubescens* | 1 | 1.02 | 1 | 0.13 | 4 | 33.33 | 1.5 | 1 | 6 | 100 | 3 | 15.4 | 16.9 | 16.17 | 80.0 | 2 | 1 | dosi | Cu | HSa | 2 | 2 | 3 | 2 |
| Rosaceae | *Eriobotrya japonica* | 1 | 1.02 | 1 | 0.13 | 4 | 33.33 | 1.5 | 1 | 5 | 83 | 3 | 11.1 | 11.1 | 11.11 | 20.0 | 2 | 1 | dosi | Cu | HSa | 1 | 1 | 1 | 1 |
| Rosaceae | *Fragaria × ananassa* | 1 | 1.02 | 1 | 0.13 | 2 | 16.67 | 1.5 | 1 | 1 | 17 | 1 | 14.3 | 14.3 | 14.29 | 8.8 | 1 | 1 | si | Fo | HSaVn | 1 | 1 | 1 | 1 |
| Rosaceae | *Prunus serotina* | 10 | 10.2 | 1 | 0.38 | 1 | 8.33 | 1.5 | 1 | 1 | 17 | 1 | 11 | 11 | 11.38 | 1.7 | 1 | 2 | dosi | Cu | HSaVn | 2 | 4 | 4 | 1 |
| Rubiaceae | *Coffea arabica* | 3 | 3.06 | 1 | 0.25 | 2 | 16.67 | 1 | 1 | 2 | 33 | 1 | 71.4 | 100 | 85.71 | 2.0 | 1 | 1 | dosi | FoReToCu | HSaVn | 1 | 1 | 2 | 2 |
| Sapotaceae | *Couepia polyandra* | 1 | 1.02 | 1 | 0.13 | 2 | 16.67 | 1.5 | 1 | 1 | 17 | 1 | 11.4 | 11.4 | 11.36 | 4.4 | 1 | 1 | do | Cu | HSaVsVn | 1 | 1 | 1 | 1 |
| Sapotaceae | *Manilkara zapota* | 1 | 1.02 | 1 | 0.13 | 3 | 25 | 1.5 | 1 | 2 | 33 | 1 | 10 | 30 | 20.00 | 70.0 | 2 | 1 | si | FoReTo | HVsVn | 1 | 1 | 3 | 4 |
| Sapotaceae | *Sideroxylon palmeri* | 1 | 1.02 | 1 | 0.13 | 4 | 33.33 | 1.5 | 1 | 4 | 67 | 8.75 | 13 | 93 | 41.72 | 577.9 | 2 | 2 | si | CuReTo | HVsVn | 2 | 3 | 4 | 2 |

**Appendix 2** Continuation…..

| **Family** | ***Species name*** | **Ecological Marix (Matrix X)** | | | | | | | | **Socioeconomic Matrix (Matrix W)** | | | | | | | | **Management Matrix (Response Matrix Y)** | | | | | | | |
| --- | --- | --- | --- | --- | --- | --- | --- | --- | --- | --- | --- | --- | --- | --- | --- | --- | --- | --- | --- | --- | --- | --- | --- | --- | --- |
|  |  | **No. Plots** | **% Plot** | **No. types Veg** | **% Veg** | **Months present** | **% Month present** | **Part Used Index** | **Life Cycle** | **No. Market** | **% Market** | **Markt Stall** | **Price min** | **Price max** | **Price Avera.** | **Vol. Sold** | **Interch-anged.** | **Origin** | **Ecolog. Status** | **Manag-ment.** | **Manag-ement System** | **Cat. Ecolog Status** | **Cat Manag** | **Cat. Manag. Sys** | **No. Uses** |
| Solanaceae | *Capsicum annuum* | 1 | 1.02 | 2 | 0.13 | 3 | 25 | 1.5 | 1 | 1 | 17 | 1 | 69 | 69 | 68.98 | 2.9 | 1 | 2 | dosi | Cu | SiSa | 2 | 1 | 3 | 1 |
| Solanaceae | *Capsicum annuum var. annuum* | 1 | 1.02 | 1 | 0.13 | 6 | 50 | 1.5 | 1 | 3 | 50 | 4 | 16.7 | 20 | 19.44 | 80.0 | 2 | 2 | si | FoReTo | SiHSa | 2 | 1 | 2 | 1 |
| Solanaceae | *Capsicum annuum* var*. aviculare* | 1 | 1.02 | 4 | 0.13 | 3 | 25 | 1.5 | 1 | 1 | 17 | 1 | 58.8 | 58.8 | 58.82 | 0.4 | 1 | 2 | ar | CuTo | SiSa | 2 | 1 | 2 | 1 |
| Solanaceae | *Capsicum pubescens* | 1 | 1.02 | 1 | 0.13 | 3 | 25 | 1.5 | 1 | 6 | 100 | 5 | 8.33 | 14.3 | 11.00 | 81.2 | 2 | 1 | si | FoReTo | Vn | 1 | 1 | 2 | 1 |
| Solanaceae | *Cestrum nocturmun* | 3 | 3.06 | 1 | 0.25 | 6 | 50 | 3 | 1 | 2 | 33 | 2 | 13.3 | 20.8 | 16.83 | 1.4 | 2 | 1 | si | CuFoTo | HVn | 1 | 1 | 3 | 3 |
| Solanaceae | *Physalis philadelphica* | 3 | 3.06 | 1 | 0.25 | 2 | 16.67 | 1.5 | 2 | 4 | 67 | 3 | 8.3 | 10 | 8.85 | 50.0 | 2 | 2 | dosi | CuFo | HSa | 2 | 2 | 3 | 3 |
| Solanaceae | *Solanaceae* sp. | 1 | 1.02 | 1 | 0.13 | 2 | 16.67 | 1.5 | 1 | 1 | 17 | 1 | 90.9 | 90.9 | 90.90 | 0.3 | 1 | 2 | do | Cu | SiHVs | 1 | 2 | 1 | 1 |
| Solanaceae | *Solanum lycopersicum* | 1 | 1.02 | 1 | 0.13 | 2 | 16.67 | 1.5 | 2 | 4 | 67 | 3 | 7 | 10 | 8.50 | 30.0 | 2 | 2 | arsi | ToRe | HSaVs | 1 | 1 | 3 | 3 |
| Solanaceae | *Solanum nigrescens* | 1 | 1.02 | 1 | 0.13 | 5 | 41.67 | 3 | 1 | 2 | 33 | 3.5 | 9.7 | 23 | 15.37 | 4.6 | 2 | 2 | do | Cu | HSa | 2 | 2 | 3 | 4 |
| Solanaceae | *Solanum* sp. | 1 | 1.02 | 1 | 0.13 | 2 | 16.67 | 3 | 2 | 1 | 17 | 1 | 90.9 | 90.9 | 90.90 | 0.3 | 1 | 2 | arsi | FoRe | HSaVs | 1 | 1 | 2 | 1 |
| Solanaceae | *Solanum tuberosum* | 1 | 1.02 | 3 | 0.13 | 4 | 33.33 | 1.5 | 1 | 2 | 33 | 1 | 11 | 11 | 11.11 | 2.3 | 2 | 1 | arsi | CuFoReTo | HSaVs | 1 | 1 | 3 | 1 |
| Solanaceae | *Solanum melongena* | 1 | 1.02 | 3 | 0.13 | 3 | 25 | 1.5 | 2 | 2 | 33 | 2.5 | 10 | 12 | 11.00 | 27.0 | 2 | 1 | dosi | CuRe | VsVn | 1 | 1 | 2 | 1 |
| Solanaceae | *Witheringia solanacea* | 1 | 1.02 | 5 | 0.13 | 2 | 16.67 | 3 | 1 | 1 | 17 | 1 | 6 | 6 | 6.04 | 0.8 | 1 | 2 | dosi | CuFoRe | HSa | 1 | 3 | 3 | 1 |
| Verbenaceae | *Lippia graveolens* | 18 | 18.4 | 1 | 0.75 | 3 | 25 | 2 | 1 | 4 | 67 | 3 | 9.1 | 83 | 40.07 | 5.5 | 2 | 2 | dosi | CuReTo | HSaVsVn | 1 | 2 | 4 | 8 |
| Vitaceae | *Vitis* sp. | 1 | 1.02 | 1 | 0.13 | 1 | 8.33 | 1.5 | 1 | 1 | 17 | 1 | 17 | 18 | 17.79 | 5.6 | 1 | 1 | dosi | CuPrFoTo | HSaVsVn | 1 | 1 | 1 | 1 |
| Zingiberaceae | *Renealmia alpinia* | 1 | 1.02 | 3 | 0.13 | 2 | 16.67 | 1.5 | 2 | 1 | 17 | 1 | 13.3 | 13.3 | 13.32 | 8.6 | 1 | 2 | doar | CuTo | SiHSaVsVn | 2 | 4 | 3 | 1 |

**Appendix 3.** Database used by Risk Index. Higher values indicated higher risk, the highest risk value is closer to 1 and the lowest one closer to 0. In those cases in which values of variables go in opposite directions (asterisk of Table 4), such as management factors, the value was subtracted to 1, in order to modify the sense.The table shows data where each variable was divided by the maximum value of its category in order to obtain data between 0 to 1. To Ecological Matrix: Veg.= No. of plots where a species was present in 98 samples. Todos los datos fueron ponderados al número uno Tem: No. the availability in months of each of the species in the market, PUI: Part used Index as Pieroni (2001),LC:Life Cycle; to Market Matrix= Mark:Market presence, Stall:No. of stall average where the plant species was present, Price, Average price, VolSell: Volume of sell, Exchange= commercialized or interchanged (barter), StEc: Ecological Status, CatStEc: Category of Ecological Status, TiMa: Management Types, CatTiMa: Category of Management Types, SiMa:Management Systems, CatMaSys:Categories of Management Systems, Uses: No. of different uses. All variables from values to numbers where change such as Appendix 4 showed.

|  |  |  | **Ecological Matrix** | | | | **Market_Matrix** | | | | | **Management Matrix** | | | | | | |
| --- | --- | --- | --- | --- | --- | --- | --- | --- | --- | --- | --- | --- | --- | --- | --- | --- | --- | --- |
| **No.** | **Family** | **Species** | **%Plot** | **%Month** | **PUI** | **LC** | **%Mark** | **%Stall** | **Price** | **VolSoldl** | **Inter** | **Ecol** | **CatEcSt** | **Manag.** | **CatMang** | **ManagSys** | **Cat.Ma Sys** | **Uses** |
| 1 | Amaranthaceae | *Amaranthus hybridus* | 0.61 | 0.2 | 1.00 | 1 | 1.00 | 0.69 | 0.06 | 0.45 | 1 | 0.50 | 0.00 | 0.50 | 0.20 | 0.30 | 0.20 | 0.25 |
| 2 | Anacardiaceae | *Cyrtocarpa procera* | 0.83 | 0.8 | 0.50 | 0.5 | 0.67 | 0.23 | 0.06 | 0.11 | 1 | 0.67 | 0.50 | 0.40 | 0.40 | 0.54 | 0.40 | 0.67 |
| 3 | Anacardiaceae | *Spondias mombin* | 0.94 | 0.7 | 0.50 | 0.5 | 0.17 | 0.11 | 0.04 | 0.01 | 1 | 0.33 | 0.00 | 0.40 | 0.60 | 0.70 | 0.60 | 0.33 |
| 4 | Anacardiaceae | *Spondias purpurea* | 0.94 | 0.8 | 0.50 | 0.5 | 0.50 | 0.51 | 0.03 | 0.11 | 0.5 | 0.67 | 0.50 | 0.47 | 0.40 | 0.54 | 0.40 | 0.08 |
| 5 | Araceae | *Spathiphyllum cochlearispathum* | 0.94 | 0.9 | 0.25 | 1 | 0.17 | 0.11 | 0.10 | 0.00 | 0.5 | 0.67 | 0.50 | 0.40 | 0.80 | 0.47 | 0.40 | 0.08 |
| 6 | Arecaceae | *Chamaedorea tepejilote* | 0.78 | 0.8 | 0.50 | 0.5 | 0.83 | 0.57 | 0.05 | 1.00 | 1 | 0.67 | 0.50 | 0.00 | 0.80 | 0.35 | 0.20 | 0.08 |
| 7 | Asparagaceae | *Agave potatorum* | 0.22 | 0.8 | 0.25 | 0.5 | 0.50 | 0.11 | 0.05 | 0.00 | 1 | 0.67 | 0.50 | 0.40 | 0.00 | 0.47 | 0.40 | 1.00 |
| 8 | Asparagaceae | *Agave* sp.1 | 0.94 | 0.5 | 0.25 | 0.5 | 0.50 | 0.57 | 0.04 | 0.20 | 1 | 0.67 | 0.50 | 0.50 | 0.20 | 0.80 | 0.80 | 0.08 |
| 9 | Asparagaceae | *Agave* sp.2 | 0.94 | 0.6 | 0.25 | 0.5 | 0.67 | 0.57 | 0.05 | 0.07 | 1 | 0.67 | 0.50 | 0.50 | 0.20 | 0.80 | 0.80 | 0.08 |
| 10 | Asparagaceae | *Yucca periculosa* | 0.83 | 0.9 | 0.25 | 0.5 | 0.50 | 0.15 | 0.05 | 0.01 | 1 | 0.67 | 0.50 | 0.45 | 0.20 | 0.60 | 0.60 | 0.58 |
| 11 | Asteraceae | *Porophyllum linaria* | 0.56 | 0.7 | 1.00 | 1 | 1.00 | 0.66 | 0.09 | 0.00 | 1 | 0.50 | 0.00 | 0.35 | 0.20 | 0.40 | 0.00 | 0.25 |
| 12 | Asteraceae | *Porophyllum macrocephalum* | 0.83 | 0.7 | 1.00 | 1 | 1.00 | 0.69 | 0.10 | 0.04 | 1 | 0.50 | 0.00 | 0.40 | 0.00 | 0.34 | 0.40 | 0.25 |
| 13 | Cactaceae | *Escontria chiotilla* | 0.39 | 0.5 | 0.50 | 0.5 | 0.67 | 0.57 | 0.05 | 0.19 | 1 | 0.33 | 0.00 | 0.40 | 0.00 | 0.50 | 0.20 | 0.58 |
| 14 | Cactaceae | *Ferocactus latispinus* | 0.39 | 0.8 | 1.00 | 0.5 | 0.17 | 0.11 | 0.36 | 0.00 | 0.5 | 0.67 | 0.50 | 0.40 | 0.20 | 0.50 | 0.20 | 0.33 |
| 15 | Cactaceae | *Myrtillocactus geometrizans* | 0.56 | 0.9 | 0.50 | 0.5 | 0.17 | 0.11 | 0.19 | 0.01 | 0.5 | 0.67 | 0.50 | 0.47 | 0.40 | 0.50 | 0.20 | 0.75 |
| 16 | Cactaceae | *Neobuxbaumia tetetzo* | 0.78 | 0.7 | 0.25 | 0.5 | 0.33 | 0.11 | 0.14 | 0.04 | 0.5 | 0.67 | 0.50 | 0.47 | 0.40 | 0.60 | 0.60 | 0.50 |
| 17 | Cactaceae | *Pachycereus weberi* | 0.39 | 0.8 | 0.33 | 0.5 | 0.17 | 0.11 | 0.50 | 0.00 | 0.5 | 0.67 | 0.50 | 0.70 | 0.60 | 0.60 | 0.60 | 0.58 |
| 18 | Cactaceae | *Stenocereus pruinosus* | 0.72 | 0.8 | 0.50 | 0.5 | 0.50 | 1.03 | 0.06 | 0.71 | 1 | 0.33 | 0.00 | 0.40 | 0.20 | 0.40 | 0.00 | 0.58 |

**Appendix 3** Continued.

|  |  |  | **Ecological Matrix** | | | | **Market_Matrix** | | | | | **Management Matrix** | | | | | | |
| --- | --- | --- | --- | --- | --- | --- | --- | --- | --- | --- | --- | --- | --- | --- | --- | --- | --- | --- |
| **No.** | **Family** | **Species** | **%Plot** | **%Month** | **PUI** | **LC** | **%Mark** | **%Stall** | **Price** | **VolSoldl** | **Inter** | **Ecol** | **CatEcSt** | **Manag.** | **CatMang** | **ManagSys** | **Cat.Ma Sys** | **Uses** |
| 19 | Cactaceae | *Stenocereus stellatus* | 0.44 | 0.7 | 0.50 | 0.5 | 0.67 | 0.57 | 0.04 | 0.62 | 1 | 0.33 | 0.00 | 0.40 | 0.20 | 0.50 | 0.20 | 0.67 |
| 20 | Chenopodiaceae | *Chenopodium ambrosioides* | 0.94 | 0 | 1.00 | 1 | 1.00 | 0.34 | 0.05 | 0.04 | 1 | 0.50 | 0.00 | 0.45 | 0.20 | 0.40 | 0.40 | 0.33 |
| 21 | Chenopodiaceae | *Chenopodium berlandieri* | 0.94 | 0.7 | 0.25 | 1 | 0.17 | 0.11 | 0.01 | 0.01 | 0.5 | 0.33 | 0.50 | 0.30 | 0.60 | 0.20 | 0.60 | 0.17 |
| 22 | Cucurbitaceae | *Apodanthera aspera* | 0.94 | 0.8 | 0.33 | 0.5 | 0.17 | 0.11 | 1.00 | 0.00 | 0.5 | 0.50 | 0.00 | 0.47 | 0.40 | 0.50 | 0.60 | 0.08 |
| 23 | Cucurbitaceae | *Cucurbita mostacha* | 0.94 | 0.8 | 0.33 | 1 | 0.17 | 0.11 | 0.04 | 0.01 | 0.5 | 0.00 | 0.50 | 0.00 | 0.80 | 0.30 | 0.60 | 0.25 |
| 24 | Cucurbitaceae | *Cucurbita pepo* | 0.78 | 0.7 | 0.25 | 1 | 1.00 | 0.51 | 0.04 | 0.14 | 1 | 0.00 | 0.50 | 0.00 | 0.80 | 0.20 | 0.40 | 0.33 |
| 25 | Cucurbitaceae | *Sechium edule* | 0.94 | 0.3 | 1.00 | 1 | 1.00 | 0.57 | 0.03 | 0.15 | 1 | 0.33 | 0.00 | 0.00 | 0.80 | 0.30 | 0.60 | 0.08 |
| 26 | Dioscoreaceae | *Dioscorea* sp. | 0.94 | 0.8 | 0.33 | 0.5 | 0.17 | 0.11 | 0.04 | 0.01 | 0.5 | 0.67 | 0.50 | 0.60 | 0.40 | 0.80 | 0.80 | 0.17 |
| 27 | Euphorbiaceae | *Jatropha neopauciflora* | 0.94 | 0.7 | 0.33 | 0.5 | 0.33 | 0.17 | 0.26 | 0.01 | 1 | 0.67 | 0.50 | 0.47 | 0.40 | 0.80 | 0.80 | 0.17 |
| 28 | Fabaceae | *Crotalaria pumila* | 0.94 | 0.7 | 1.00 | 1 | 0.17 | 0.11 | 0.01 | 0.01 | 0.5 | 0.50 | 0.00 | 0.60 | 0.60 | 0.40 | 0.40 | 0.33 |
| 29 | Fabaceae | *Inga vera* | 0.94 | 0.7 | 0.50 | 0.5 | 0.50 | 0.17 | 0.03 | 0.01 | 0.5 | 0.00 | 0.50 | 0.00 | 0.80 | 0.50 | 0.20 | 0.08 |
| 30 | Fabaceae | *Leucaena esculenta* | 0.94 | 0.7 | 0.50 | 0.5 | 1.00 | 0.38 | 0.06 | 0.11 | 1 | 0.17 | 0.00 | 0.30 | 0.60 | 0.40 | 0.00 | 0.67 |
| 31 | Fabaceae | *Leucaena leucocephala* | 0.94 | 0.7 | 0.50 | 0.5 | 0.50 | 0.29 | 0.07 | 0.00 | 1 | 0.33 | 0.00 | 0.30 | 0.20 | 0.50 | 0.20 | 0.58 |
| 32 | Fabaceae | *Phaseolus coccineus* | 0.89 | 0.8 | 0.50 | 1 | 0.83 | 0.40 | 0.04 | 0.08 | 1 | 0.33 | 0.00 | 0.00 | 0.80 | 0.40 | 0.40 | 0.25 |
| 33 | Fabaceae | *Phaseolus* sp. | 0.94 | 0.8 | 0.25 | 1 | 0.33 | 0.29 | 0.03 | 0.01 | 0.5 | 0.33 | 0.00 | 0.00 | 0.80 | 0.20 | 0.60 | 0.08 |
| 34 | Fabaceae | *Phaseolus vulgaris* | 0.89 | 0.6 | 0.33 | 1 | 0.83 | 0.23 | 0.04 | 0.23 | 0.5 | 0.33 | 0.00 | 0.00 | 0.80 | 0.20 | 0.40 | 0.17 |
| 35 | Fabaceae | *Pithecellobium dulce* | 0.89 | 0.9 | 0.50 | 0.5 | 0.17 | 0.11 | 0.05 | 0.01 | 0.5 | 0.33 | 0.00 | 0.47 | 0.40 | 0.50 | 0.20 | 1.00 |
| 36 | Lamiaceae | *Clinopodium mexicanum* | 0.94 | 0.8 | 1.00 | 1 | 0.67 | 0.51 | 0.04 | 0.14 | 1 | 0.67 | 0.50 | 0.33 | 0.40 | 0.50 | 0.60 | 0.17 |
| 37 | Lauraceae | *Litsea glaucescens* | 0.72 | 0.7 | 0.67 | 0.5 | 0.67 | 0.29 | 0.15 | 0.03 | 1 | 0.67 | 0.50 | 0.60 | 0.40 | 0.47 | 0.40 | 0.08 |
| 38 | Lauraceae | *Litsea neesiana* | 0.94 | 0.9 | 0.67 | 0.5 | 0.17 | 0.11 | 0.13 | 0.00 | 0.5 | 0.67 | 0.50 | 0.60 | 0.40 | 0.80 | 0.80 | 0.08 |
| 39 | Lauraceae | *Persea americana* | 0.61 | 0.7 | 0.67 | 0.5 | 1.00 | 0.46 | 0.03 | 0.06 | 1 | 0.00 | 0.50 | 0.00 | 0.80 | 0.40 | 0.40 | 0.33 |
| 40 | Malpighiaceae | *Byrsonima crassifolia* | 0.94 | 0.7 | 0.50 | 0.5 | 0.33 | 0.11 | 0.08 | 0.03 | 0.5 | 0.33 | 0.00 | 0.40 | 0.40 | 0.30 | 0.60 | 0.33 |
| 41 | Malvaceae | *Ceiba aesculifolia* subsp. *parvifolia* | 0.39 | 0.6 | 0.33 | 0.5 | 0.50 | 0.53 | 0.14 | 0.35 | 1 | 0.67 | 0.50 | 0.47 | 0.40 | 0.50 | 0.20 | 0.83 |

**Appendix 3.** Continued…

|  |  |  | **Ecological Matrix** | | | | **Market_Matrix** | | | | | **Management Matrix** | | | | | | |
| --- | --- | --- | --- | --- | --- | --- | --- | --- | --- | --- | --- | --- | --- | --- | --- | --- | --- | --- |
| **No.** | **Family** | **Species** | **%Plot** | **%Month** | **PUI** | **LC** | **%Mark** | **%Stall** | **Price** | **VolSoldl** | **Inter** | **Ecol** | **CatEcSt** | **Manag.** | **CatMang** | **ManagSys** | **Cat.Ma Sys** | **Uses** |
| 42 | Nolineaceae | *Dasylirion serratifolium* | 0.56 | 0.4 | 0.25 | 1 | 0.50 | 0.40 | 0.08 | 0.05 | 1 | 0.67 | 0.50 | 0.60 | 0.40 | 0.80 | 0.80 | 0.17 |
| 43 | Phytolaccaceae | *Phytolacca icosandra* | 0.89 | 0.8 | 1.00 | 0.5 | 0.17 | 0.11 | 0.02 | 0.00 | 0.5 | 0.50 | 0.00 | 0.60 | 0.40 | 0.40 | 0.40 | 0.08 |
| 44 | Pinaceae | *Pinus cembroides* | 0.94 | 0.7 | 0.33 | 0.5 | 0.17 | 0.11 | 0.15 | 0.03 | 0.5 | 0.67 | 0.50 | 0.00 | 0.80 | 0.60 | 0.60 | 0.08 |
| 45 | Piperaceae | *Peperomia peltilimba* | 0.94 | 0.8 | 1.00 | 0.5 | 0.17 | 0.11 | 0.13 | 0.00 | 0.5 | 0.67 | 0.50 | 0.60 | 0.40 | 0.54 | 0.40 | 0.08 |
| 46 | Portulacaceae | *Portulaca oleracea* | 0.94 | 0.6 | 0.50 | 0.5 | 1.00 | 0.34 | 0.05 | 0.12 | 1 | 0.33 | 0.00 | 0.10 | 0.60 | 0.47 | 0.40 | 0.17 |
| 47 | Rosaceae | *Prunus serotina* | 0.44 | 0.9 | 0.50 | 0.5 | 0.17 | 0.11 | 0.04 | 0.00 | 0.5 | 0.33 | 0.00 | 0.40 | 0.00 | 0.50 | 0.20 | 0.08 |
| 48 | Sapotaceae | *Sideroxylon palmeri* | 0.94 | 0.6 | 0.50 | 0.5 | 0.67 | 1.00 | 0.14 | 0.87 | 1 | 0.33 | 0.00 | 0.47 | 0.40 | 0.50 | 0.20 | 0.17 |
| 49 | Solanaceae | *Capsicum annuum* | 0.94 | 0.7 | 0.50 | 0.5 | 0.17 | 0.11 | 0.22 | 0.00 | 0.5 | 0.33 | 0.00 | 0.00 | 0.80 | 0.47 | 0.40 | 0.08 |
| 50 | Solanaceae | *Capsicum annuum* var. *annuum* | 0.94 | 0.4 | 0.50 | 0.5 | 0.50 | 0.46 | 0.06 | 0.12 | 1 | 0.33 | 0.00 | 0.00 | 0.80 | 0.30 | 0.60 | 0.08 |
| 51 | Solanaceae | *Capsicum annuum* var. *aviculare* | 0.94 | 0.7 | 0.50 | 0.5 | 0.17 | 0.11 | 0.19 | 0.00 | 0.5 | 0.33 | 0.00 | 0.00 | 0.80 | 0.30 | 0.60 | 0.08 |
| 52 | Solanaceae | *Physalis philadelphica* | 0.83 | 0.8 | 0.50 | 1 | 0.67 | 0.34 | 0.03 | 0.08 | 1 | 0.17 | 0.00 | 0.10 | 0.60 | 0.27 | 0.40 | 0.25 |
| 53 | Solanaceae | *Solanaceae* sp. | 0.94 | 0.8 | 0.50 | 0.5 | 0.17 | 0.11 | 0.29 | 0.00 | 0.5 | 0.33 | 0.50 | 0.70 | 0.60 | 0.40 | 0.80 | 0.08 |
| 54 | Solanaceae | *Solanum lycopersicum* | 0.94 | 0.8 | 0.50 | 1 | 0.67 | 0.34 | 0.03 | 0.05 | 1 | 0.00 | 0.50 | 0.00 | 0.80 | 0.27 | 0.40 | 0.25 |
| 55 | Solanaceae | *Solanum nigrescens* | 0.94 | 0.5 | 1.00 | 0.5 | 0.33 | 0.40 | 0.05 | 0.01 | 1 | 0.50 | 0.00 | 0.70 | 0.60 | 0.40 | 0.40 | 0.33 |
| 56 | Solanaceae | *Solanum* sp. | 0.94 | 0.8 | 1.00 | 1 | 0.17 | 0.11 | 0.29 | 0.00 | 0.5 | 0.33 | 0.50 | 0.80 | 0.80 | 0.30 | 0.60 | 0.08 |
| 57 | Solanaceae | *Witheringia solanacea* | 0.94 | 0.8 | 1.00 | 0.5 | 0.17 | 0.11 | 0.02 | 0.00 | 0.5 | 0.67 | 0.50 | 0.60 | 0.40 | 0.20 | 0.40 | 0.08 |
| 58 | Verbenaceae | *Lippia graveolens* | 0.94 | 0.7 | 0.67 | 0.5 | 0.67 | 0.34 | 0.13 | 0.01 | 1 | 0.67 | 0.50 | 0.40 | 0.60 | 0.50 | 0.20 | 0.67 |
| 59 | Zingiberaceae | *Renealmia alpinia* | 0.94 | 0.8 | 0.50 | 1 | 0.17 | 0.11 | 0.04 | 0.01 | 0.5 | 0.17 | 0.00 | 0.45 | 0.20 | 0.47 | 0.40 | 0.08 |

**Appendix 4.** Description of partial canonical analysis variables used in the database of 105 edible plant species. M: Matrices: 1 = Managements variables (Matrix Y), 2=Ecological Variables (Matrix X), 3=Socioeconomic variables (Matrix W).

| **M** | **Variable** | **Description** |
| --- | --- | --- |
| 1 | Ecological Status | Conditions of habitat of a plant species, wild=1, weed or ruderal =2, domesticated=3. To obtained a number each category was considered, e.i. a species plant weed (2) and wild(1), its value was: 2+1=3 |
| 1 | Categories of Ecological Status | Numbers of categories were summed, by example when a plant had two categories those were summed, such as wild and ruderal, two categories were 2. |
| 1 | Management types | Characteristics of the management, is a gradient of complexity, if it´s recollected (1), tolerated (2), promoted (3), protected (4) or cultivated (5). Each species plant was considered as a number. When a species plant had 2 o more categories those were summed, such as, tolerated (2) and promoted (3), the result was 2+3=5. |
| 1 | Categories of management types | Numbers of categories were summed, by example when a plant had two categories those were summed, such as recollected plus protected, two categories, were 2. |
| 1 | Management systems | Spatial system where a plant is present, since natural vegetation (1), secondary vegetation (2), agroforestal system (3), homegardens (4), intensive system (5). When a species plant had more than one categories those were summed, i.e. : natural vegetation (1), homegardens (4)= 1+4 =5. |
| 1 | Categories of management system | Number of categories were summed, by example when a plant had two categories, those were summed, such as present in homegarden plus agroforestal system, in total we have 2 categories |
| 1 | Origin | We considered as native plants those with natural populations in the arid and semiarid region of the Valley and in the “Sierra Negra” of Puebla. Plants species that come from other Mexican regions and other parts of the world were considered as introduced. Native was 2, introduced=1 |
| 1 | Uses | Number of uses considered on database of Blancas et al 2010 and bibliographic information.When we obtained more than one value those were summed. |
| 2 | Percentage presence in plots | Number of plots where a species was present obtained through revision of its presence in 98 samples. Each cell value was divided between 98 and after we calculated its percentage. |
| 2 | Percentage types of vegetation | 6 types of vegetation of Valiente-Banuet 2000 were considered, plus agricultural. For each species plant was registered in how many types of vegetation are possible to find it and this number was divided between seven. After that, its percentage respect to total was calculated. |

**Appendix 4.** Continuation…

| **N** | **Variable** | **Description** |
| --- | --- | --- |
| 2 | Percentage months | Was calculated from the availability in months of each of the species in the market. We considered twelve months, so that the value of each species was divided by 12 and then was calculated their percentage. |
| 2 | Part used index | Was based on Pieroni (2001) whom has a numerical scale. |
| 2 | Life cycle | We aggregated a number when a species plant was 1=annual, and a 2= perennial. |
| 3 | Percentage Markets | Number of markets where a species plant was found, maximum 6 markets, minimum 1 market. Each specie value was divided between maximum (6) and its percentage was calculated. |
| 3 | Average Market Stall | Number of stalls where a species was found in each market was summed. Total number of stall was divided between numbers of total stalls to obtain the average by market. Total markets stall were summed and its average was obtained. |
| 3 | Average price | Number of stalls where a species was found in each market were summed. Total numbers of stalls were divided between numbers of total stalls to obtain the average of stalls from a market. Total markets stalls were summed and its average was obtained. |
| 3 | Sales volume | We summed total sales for all the markets in the same week or month. Average was conducted when we had information of two or more weeks or months. |
| 3 | Interchanged ways | We considered three types of interchanged of products, 1=interchanged just by money, 2= interchanged by corn and or by money, 3=interchanged just by corn. |

**Appendix 5.** Description of partial canonical analysis variables used in the database of 59 edible plant species. F factor 1: 1 = Manag~~e~~ment, 2=Ecological, 3=Socioeconomic. Risk index were applied to this data. Data were considered found a gradient from low to high risk, by that we changed the scale of some factors such as these with an asterisk. We expected to construct a risk index which higher values would indicate higher risk. In those cases where values of variables that go in opposite direction, such as management factors, the values were substracted to 1, in order to change the range.

| **F** | **Variable** | **Description** |
| --- | --- | --- |
| 1 | Ecological Status* | Conditions of habitat of a plant species, wild=1, weed or ruderal =2, domesticated=3. To obtained a number each category was considered, e.i. a species plant weed (2) and wild(1), its value was: 2+1=3 |
| 1 | Categories of Ecological Status | Numbers of categories were summed, by example when a plant had two categories those were summed, such as wild and ruderal, two categories were 2. |
| 1 | Management types* | Characteristics of the management, is a gradient of complexity, if it´s recollected (1), tolerated (2), promoted (3), protected (4) or cultivated (5). Each species plant was considered as a number. When a species plant had 2 o more categories those were summed, such as, tolerated (2) and promoted (3), the result was 2+3=5. |
| 1 | Categories of management types | Numbers of categories were summed, by example when a plant had two categories those were summed, such as recollected plus protected, two categories, were 2. |
| 1 | Management systems* | Spatial system where a plant is present, since natural vegetation (1), secondary vegetation (2), agroforestal system (3), homegardens (4), intensive system (5). When a species plant had more than one categories those were summed, i.e. : natural vegetation (1), homegardens (4)= 1+4 =5. |
| 1 | Categories of management system | Number of categories were summed, by example when a plant had two categories, those were summed, such as present in homegarden plus agroforestal system, in total we have 2 categories |
| 1 | Uses* | Number of uses considered on database of Blancas et al 2010 and bibliographic information.When we obtained more than one value those were summed. |
| 2 | Percentage presence in plots* | Number of plots where a species was present obtained through revision of its presence in 98 samples. Each cell value was divided between 98 and after we calculated its percentage. |

**Appendix 5.** Continuation…

| **F** | **Variable** | **Description** |
| --- | --- | --- |
| 2 | Percentage months* | Was calculated from the availability in months of each of the species in the market. We considered twelve months, so that the value of each species was divided by 12 and then was calculated their percentage. |
| 2 | Part used index | Was based on Pieroni (2001) whom has a numerical scale. |
| 2 | Life cycle | We aggregated a number when a species plant was 1=annual, and a 2= perennial. |
| 3 | Percentage Markets | Number of markets where a species plant was found, maximum 6 markets, minimum 1 market. Each specie value was divided between maximum (6) and its percentage was calculated. |
| 3 | Average Market Stall | Number of stalls where a species was found in each market was summed. Total number of stall was divided between numbers of total stalls to obtain the average by market. Total markets stall were summed and its average was obtained. |
| 3 | Average price* | Number of stalls where a species was found in each market were summed. Total numbers of stalls were divided between numbers of total stalls to obtain the average of stalls from a market. Total markets stalls were summed and its average was obtained. |
| 3 | Sales volume | We summed total sales for all the markets in the same week or month. Average was conducted when we had information of two or more weeks or months. |
| 3 | Interchanged ways* | We considered three types of interchanged of products, 1=interchanged just by money, 2= interchanged by corn and or by money, 3=interchanged just by corn. |
